# The Chemistry and the Anti-Inflammatory Activity of Polymethoxyflavonoids from *Citrus* Genus

**DOI:** 10.3390/antiox12010023

**Published:** 2022-12-22

**Authors:** Gianfranco Fontana, Maurizio Bruno, Francesco Sottile, Natale Badalamenti

**Affiliations:** 1 Dipartimento di Scienze e Tecnologie Biologiche, Chimiche e Farmaceutiche (STEBICEF), Università Degli Studi di Palermo, Viale delle Scienze Ed. 17, 90128 Palermo, Italy; 2 Dipartimento di Architettura, Università Degli Studi di Palermo, Centro di Conservazione della Biodiversità di Interesse Agrario, Viale delle Scienze Ed. 14, 90128 Palermo, Italy

**Keywords:** *Citrus* genus, polymethoxyflavonoids, hydroxylated polymethoxyflavonoids, polyphenols, NMR, anti-inflammatory activity

## Abstract

Polymethoxyflavonoids (PMFs) are a large group of compounds belonging to the more general class of flavonoids that possess a flavan carbon framework decorated with a variable number of methoxy groups. Hydroxylated polymethoxyflavonoids (HPMFs), instead, are characterized by the presence of both hydroxyl and methoxy groups in their structural unities. Some of these compounds are the aglycone part in a glycoside structure in which the glycosidic linkage can involve the −OH at various positions. These compounds are particular to *Citrus* genus plants, especially in fruits, and they are present mainly in the peel. A considerable number of PMFs and HPMFs have shown promising biological activities and they are considered to be important nutraceuticals, responsible for some of the known beneficial effects on health associated with a regular consumption of *Citrus* fruits. Among their several actions on human health, it is notable that the relevant contribution in controlling the intracellular redox imbalance is associated with the inflammation processes. In this work, we aim to describe the status concerning the chemical identification and the anti-inflammatory activity of both PMFs and HPMFs. In particular, all of the chemical entities unambiguously identified by isolation and complete NMR analysis, and for which a biochemical evaluation on the pure compound was performed, are included in this paper.

## 1. Introduction

The large group referred to as the genus *Citrus* has been the subject of multiple studies to determine its origin and spread [1]. There is a general agreement that the primary area of origin is identified in the downstream area of the Himalayan mountainous complex, in an area between northeastern India and southwestern China. In the remote area of Meghalaya, an Indian area bordering China, there are still some neglected *Citrus* able to demonstrate the presence of citrus in the domestication phase of the species [2]. Much has been discussed about the phylogeny of citrus, and this has also resulted in a complex botanical classification that has been modified several times over time. 

The contribution of molecular techniques has allowed for significant advances on the definition of the origin and distribution of *Citrus* by highlighting probable processes of hybridization and mutations that have fostered the current diversification of species and varieties [3]. Today, there is molecular-based scientific evidence that allows us to consider citron (*Citrus medica*), mandarin (*Citrus reticulata*), and pomelo (*Citrus maxima*) as the progenitor species from which all other cultivated citrus species then originated in different ways, forms and at different times [4].

The great plasticity of adaptation of the species has allowed its presence in many countries of the world with a great spread in cultivation, mainly as oranges, mandarins, lemons, and grapefruits [5]. That is because of their well-known sensory characteristics and widespread therapeutic and nutritional properties, owing to the presence of nutrients and biologically active compounds providing sugars, volatiles, organic acids (citric acid), dietary metabolites, amino acids, fibers, vitamin B6, vitamin C, and macro- and micronutrients, determining that citrus represent the most produced, consumed, and processed fruits globally [6]. 

The latest official data [7] report over 140 million tons with about 50% grown in the Asian continent and 14% in Brazil. Spain, Egypt, and Turkey are the main producing countries in the Mediterranean whereas on the African continent, South Africa contributes to about 2% of world production. The sweet orange (*C. sinensis*) is the world’s most produced citrus fruit and accounts for about half of global production (50%), followed by the tangerine (*C. reticulata*), lemon (*C. limon*), and grapefruit (*C. paradisi*). Approximately 75% of the production is usually consumed as fresh fruit, whereas the remaining 25% is processed to produce juices, jams, and other by-products from whose processing, moreover, several secondary products are obtained [8,9]. 

Furthermore, and not to be overlooked, is the contribution of the production of citrus plants for ornamental purposes, which has now assumed a significant role in the sector with substantial volumes moving from the Mediterranean Basin to within Europe and the Arabian Peninsula [10].

Phytochemically, the fruits of the genus *Citrus* are characterized by the massive presence of sugars, especially polysaccharides, vitamins, and mineral salts, and by smaller quantities of protein and lipid compounds. Chemically, the colour of the fruit depends on both carotenoids and different flavonoids, whereas the limonoids are responsible for the bitter taste of the juice. Terpenoids, on the other hand, are the class of chemical constituents responsible for the aroma [11,12].

**Figure 1:**
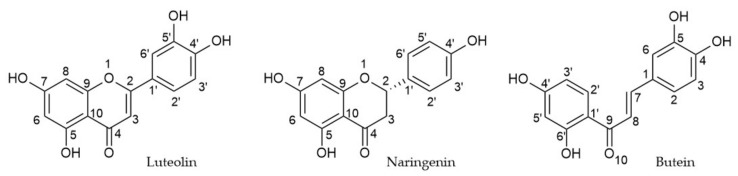
Chemical structure of flavone (e.g., luteolin), flavanone (e.g., naringenin), and chalcone (e.g., butein).

In particular, flavonoids are a wide class of phytochemicals most often extracted from the seeds, roots, leaves, and fruits peels of *Citrus* plants. Table 1, Table 2 and Table 3 report the botanical origin of compounds **1–79**. Regarding the basic structure of their carbon framework, it is possible to distinguish three main categories: flavones (2-aryl-benzopyran-4-ones) (e.g., luteolin; Figure 1), flavanones ((*S*)-2-aryl-chroman-4-ones) (e.g., naringenin; Figure 1), and chalcones (1,3-diphenyl-2*E*-propene-1-one) (e.g., butein; Figure 1). These fundamental skeletons are decorated with several phenolic hydroxyl groups. A variable number of the −OH groups can be converted in vivo to the corresponding methyl ethers or to glycosides in which sugar moieties are attached by a glycosyl linkage to one of the several hydroxy groups. 

Although there are different reviews that have extensively investigated the biosynthetic aspects of flavonoids from the *Citrus* genus [13,14] and their generic biological aspects [15], with a focus on the antioxidant properties of pure flavones extracted [16], this review aims to cover the literature concerning the spectroscopical properties and the anti-inflammatory activity of *Citrus* polymethoxyflavonoids (PMFs) that are chemical entities bearing only methoxy groups as substituents, and hydroxylated polymethoxyflavonoids (HPMFs) in which more than the one hydroxyl function is converted to a methoxy one.

**Table 1:** Polymethoxyflavones identified in the genus *Citrus*.

No.	Compound	Species	References
**1**	2′,3′-Dimethoxyflavone	Brazilian orange essential oil	[17]
**2**	2′,4′-Dimethoxyflavone	Brazilian orange essential oil	[17]
**3**	3′,4′-Dimethoxyflavone	*C. platymamma*	[18]
**4**	5,4′-Dimethoxyflavone	Brazilian orange essential oil	[17]
**5**	5,7-Dimethoxyflavone (Chrysin dimethyl ether)	Brazilian orange essential oil	[17]
**6**	7,4′-Dimethoxyflavone	Brazilian orange essential oil	[17]
	3′,4′-Dihydroxy-7,5′-dimethoxyflavone	*C. reticulata; C. sinensis*	[19]
**7**	5,6-Dihydroxy-7,4′-dimethoxyflavone (Ladanein)	Fructus Aurantii (*C. aurantium* dried)	[20]
**8**	3,5,6-Trihydroxy-7,4′-dimethoxyflavone	*C. medica*	[21]
**9**	5,7,4′-Trihydroxy-6,3′-dimethoxyflavone (Jaceosidin)	*C. hassaku*	[20]
**10**	5,7,4′-Trihydroxy-6,8-dimethoxyflavone (Demethoxysudachitin)	*C. sudachi*	[22]
	3,5,7,4′-Tetrahydroxy-8,3′-dimethoxyflavone	*C. unshiu*	[23]
	5,7,3′,4′-Tetrahydroxy-8,3′-dimethoxyflavone	*C. sudachi*	[24]
**11**	2′,3′,4′-Trimethoxyflavone	Brazilian orange essential oil	[17]
**12**	3,5,7-Trimethoxyflavone (Galangin trimethyl ether)	Brazilian orange essential oil	[17]
**13**	3′,4′,5′-Trimethoxyflavone	Brazilian orange essential oil	[17]
**14**	5,7,4′-Trimethoxyflavone (Apigenin trimethyl ether)	*C. rutaceae*	[25]
**15**	5,3′,4′-Trimethoxyflavone	Brazilian orange essential oil	[17]
**16**	6,2′,3′-Trimethoxyflavone	Brazilian orange essential oil	[17]
	3-Hydroxy-5,7,8-trimethoxyflavone	*C. reticulata*; *C. sinensis*	[19]
**17**	5-Hydroxy-6,7,4′-trimethoxyflavone (Salvigenin)	*C. sinensis*	[20]
	5-Hydroxy-7,3′,4′-trimethoxyflavone	*C. reticulata*; *C. sinensis*	[19]
**18**	5-Hydroxy-7,8,4′-trimethoxyflavone (Isoscutellarein 7,8,4′-trimethyl ether)	*C. reticulata*	[26]
**19**	8-Hydroxy-5,7,4′-trimethoxyflavone	*C. reticulata* Blanco	[27]
	4′-Hydroxy-5,6,7-trimethoxyflavone	*C. aurantium*	[28]
**20**	5,4′-Dihydroxy-6,7,8-trimethoxyflavone (Xanthomicrol)	*C. reticulata*	[29]
**21**	3,5,6-Trihydroxy-7,3′,4′-trimethoxyflavone	*C. medica*	[21]
**22**	5,7,4′-Trihydroxy-6,8,3′- trimethoxyflavone	*C. sudachi*	[24]
**23**	3,5,7,4′-Tetrahydroxy-6,8,3′-trimethoxyflavone (Limocitrol)	*C. limon*	[30]
**24**	3,6,7,4′-Tetramethoxyflavone	*C. hallabong* (hybrid)	[31]
**25**	5,6,7,4′-Tetramethoxyflavone (Scutellarein tetramethyl ether)	*C. sinensis*; *C. reticulata*	[20]
**26**	5,7,8,4′-Tetramethoxyflavone (6-Demethoxytangeretin)	*C. reticulata* Blanco cv. Ponkan	[20]
**27**	5,7,3′,4′-Tetramethoxyflavone (Luteolin tetramethyl ether)	*C. reticulata*; *C. sinensis*	[19]
**28**	6,7,8,4′-Tetramethoxyflavone	*C. unshiu*	[32]
	7,8,3′,4′-Tetramethoxyflavone	*C. platymamma*	[18]
**29**	7,3′,4′,5′-Tetramethoxyflavone	*C. reticulata*	[33]
**30**	3-Hydroxy-5,6,7,4′-tetramethoxyflavone (Eupatorin-5-methylether)	*C. sinensis*	[34]
	5-Hydroxy-3,6,7,8-tetramethoxyflavone	*C. reticulata*; *C. sinensis*	[19]
**31**	5-Hydroxy-3,6,7,4′-tetramethoxyflavone (Penduletin 4′-methyl ether)	*C. reticulata*	[27]
**32**	5-Hydroxy-3,7,8,4′-tetramethoxyflavone	*C. albanicus*; *C. parviflorus*	[35]
**33**	5-Hydroxy-3,7,3′,4′-tetramethoxyflavone (Retusin)	*C. miaray*	[36]
**34**	5-Hydroxy-6,7,8,4′-tetramethoxyflavone (Gardenin B)	*Fructus aurantii* (*C. aurantium* dried)	[37]
**35**	5-Hydroxy-6,7,3′,4′-tetramethoxyflavone (5-Desmethylsinensetin)	*C. aurantium*	[28]
	5-Hydroxy-7,8,3′,4′-tetramethoxyflavone	*C. reticulata*; *C. sinensis*	[19]
**36**	6-Hydroxy-5,7,8,4′-tetramethoxyflavone	*C. reticulata*; *C. sinensis*	[19]
**37**	7-Hydroxy-5,6,8,4′-tetramethoxyflavone	*C. reticulata*; *C. sinensis*	[19]
	7-Hydroxy-5,6,3′,4′-tetramethoxyflavone	*C. reticulata*	[38]
**38**	4′-Hydroxy-5,6,7,8-tetramethoxyflavone	*C. reticulata*	[26]
**39**	5,7-Dihydroxy-6,8,3′,4′-tetramethoxyflavone (Hymenoxin)	*C. medica* L.	[21]
	5,8-Dihydroxy-3,7,3′,4′-tetramethoxyflavone	*C. miaray*	[36]
**40**	5,3′-Dihydroxy-3,7,4′,5′-tetramethoxyflavone	*C. monspeliensis*	[39]
**41**	5,4′-Dihydroxy-6,7,8,3′-tetramethoxyflavone (8-Methoxycirsilineol)	*C. reticulata*	[29]
	7,4′-Dihydroxy-5,6,8,3′-tetramethoxyflavone	*C. deliciosa*	[40]
**42**	3,5,6,8,4′-Pentamethoxyflavone	*C. reticulata*	[41]
**43**	3,5,7,3′,4′-Pentamethoxyflavone (Quercetin pentamethyl ether)	*C. miaray*	[20]
	3,6,7,8,4′-Pentamethoxyflavone	*C. sinensis*	[20]
**44**	5,6,7,8,4′-Pentamethoxyflavone (Tangeretin)	*C. sinensis*	[20]
**45**	5,6,7,3′,4′-Pentamethoxyflavone (Sinensitin)	*C. reticulata*; *C. sinensis*	[19]
**46**	5,7,8,3′,4′-Pentamethoxyflavone (Isosinensetin)	*C. sinensis*	[20]
**47**	5,7,2′,3′,4′-Pentamethoxyflavone	*C. reticulata* Blanco; *C. reticulata* Chachi	[42]
**48**	6,7,8,3′,4′-Pentamethoxyflavone (Demethylnobiletin)	*C. reticulata* Blanco cv. Ponkan	[20]
**49**	3-Hydroxy-5,6,7,8,4′-pentamethoxyflavone (3-Demethylnobiletin)	*C. sinensis* Osbeck	[34]
**50**	5-Hydroxy-3,6,7,8,4′-pentamethoxyflavone (5-Hydroxyauranetin)	*C. aurantium*	[43]
**51**	5-Hydroxy-3,7,8,3′,4′-pentamethoxyflavone (Gossypetin pentamethylether)	*C. sinensis* Osbeck	[20]
**52**	5-Hydroxy-6,7,8,3′,4′-pentamethoxyflavone (5-Demethylnobiletin)	*C. sinensis*	[34]
**53**	5-Hydroxy-6,7,3′,4′,5′-pentamethoxyflavone (Umuhengerin)	*C. reticulata*; *C. sinensis*	[19]
**54**	7-Hydroxy-3,5,6,3′,4′-pentamethoxyflavone	*C. reticulata*	[44]
**55**	7-Hydroxy-5,6,8,3′,4′-pentamethoxyflavone (7-Demethylnobiletin)	*C. reticulata*; *C. sinensis*	[19]
**56**	8-Hydroxy-3,5,6,7,4′-pentamethoxyflavone	*C. aurantifolia*	[45]
**57**	3′-Hydroxy-5,6,7,8,4′-pentamethoxyflavone (3-Hydroxytangeretin)	*C. changshan-huyou*	[46]
**58**	4′-Hydroxy-5,6,7,8,3′-pentamethoxyflavone (4′-Demethylnobiletin)	*C. reticulata*; *C. sinensis*	[19]
**59**	3,5,6,7,8,4′-Hexamethoxyflavone (3-Methoxytangeretin)	*C. sinensis*	[20]
	3,5,6,7,3′,4′-Hexamethoxyflavone	Commercially *Citrus* peels extract	[47]
	3,5,6,8,3′,4′-Hexamethoxyflavone	*C. hassaku*	[20]
**60**	3,5,7,8,2′,5′-Hexamethoxyflavone	*C. reticulata* Blanco	[48]
**61**	3,5,7,8,3′,4′-Hexamethoxyflavone (Gossypetin hexamethyl ether)	*C. hassaku*	[20]
**62**	3,6,7,8,2′,5′-Hexamethoxyflavone	*Citrus unshiu*	[32]
**63**	5,6,7,8,3′,4′-Hexamethoxyflavone (Nobiletin)	*C. reticulata*	[44]
**64**	5,6,7,3′,4′,5′-Hexamethoxyflavone	*C. reticulata*; *C. sinensis*	[19]
**65**	5,7,8,3′,4′,5′-Hexamethoxyflavone (Bannamurpanisin)	*C. reticulata*; *C. sinensis*	[19]
**66**	3-Hydroxy-5,6,7,8,3′,4′-hexamethoxyflavone (Natsudaidain)	*C. aurantium*	[28]
**67**	5-Hydroxy-3,6,7,8,3′,4′-hexamethoxyflavone	*C. kinokuni*	[20]
**68**	5-Hydroxy-6,7,8,3′,4′,5′-hexamethoxyflavone (Gardenin A)	*C. reticulata*; *C. sinensis*	[19]
	6-Hydroxy-3,5,7,8,3′,4′-hexamethoxyflavone	*C. unshiu*	[23]
**69**	7-Hydroxy-3,5,6,8,3′,4′-hexamethoxyflavone	*C. reticulata*	[44]
**70**	8-Hydroxy-3,5,6,7,3′,4′-hexamethoxyflavone	*C. aurantifolia*	[45]
	8-Hydroxy-5,6,7,3′,4′,5′-hexamethoxyflavone	*C. reticulata*; *C. sinensis*	[19]
	3,5,6,7,8,3′,4′-Heptamethoxyflavone	*C. miaray*	[36]
**71**	5,6,7,8,3′,4′,5′-Heptamethoxyflavone	Brazilian orange essential oil	[49]

**Table 2:** Polymethoxyflavanones identified in the genus *Citrus*.

No.	Compound	Species	References
**72**	5,6,7,4′-Tetramethoxyflavanone	*C. sinensis*	[34]
	5-Hydroxy-3,6,7,4′-tetramethoxyflavanone	*C. reticula* Blanco	[50]
**73**	5,6,7,8,4′-Pentamethoxyflavanone	*C. reticula* Blanco	[50]
**74**	5,6,7,3′,4′-Pentamethoxyflavanone	*C. reticulata*; *C. sinensis*	[19]
**75**	5,7,8,3′,4′-Pentamethoxyflavanone	*C. reticula* Blanco	[27]
	6,7,8,3′,4′-Pentamethoxyflavanone	*C. reticula* Blanco	[50]
**76**	5-Hydroxy-6,7,8,3′,4′-pentamethoxyflavanone (5-Demethylcitromitine)	*C. sinensis*	[34]
	6-Hydroxy-5,7,8,3′,4′-pentamethoxyflavanone	*C. jambhiri*	[51]
**77**	5,6,7,8,3′,4′-Hexamethoxyflavanone (Citromitin)	*C. miaray*	[36]

**Table 3:** Polymethoxychalcones identified in the *Citrus* genus.

No.	Compound	Species	References
**78**	2′-Hydroxy-3,4,4′,5′,6′-pentamethoxychalcone	*C. sinensis*	[20]
**79**	2′-Hydroxy-3,4,3′,4′,5′,6′-hexamethoxychalcone	*C. sinensis*	[34]

Properly speaking, compounds characterized by the presence of just one methoxy group do not belong to the general type of HPMFs, and so they will be considered out of the scope of this paper. Further, we intend to report compounds with a chemical structure that has been unambiguously characterized by spectroscopical methods and that have not been discussed in previous reviews covering the topic. An update regarding molecules that have already been reviewed in the recent past is included.

An important criterion of the selection of the references reviewed within this work is also the presence of a defined relationship between bioactivity and the individual compounds. Reports concerning the bioactivity of mixtures (e.g., plant extracts) have not been taken into consideration, even where these are accompanied by a chemical identification of the mixture. This is because they lack any cause–effect evidence. 

The biological, pharmacological, chemo-preventive, and therapeutic activities, as well as the molecular details of the mechanism of action of PMFs and HPMFs, have been described in several works [52,53,54,55,56]. Many of these molecules have been considered as powerful chemo-preventive agents for various important diseases, including cancer and neurodegenerative pathologies, as well as other disorders related to inflammation. Some of these compounds are rather common and ubiquitous in nature, and they have received a great deal of attention from natural product researchers; these include tangeretin (**44**) [57,58], nobiletin (**63**) [57,59,60,61], sinensitin (**45**) [62], and jaceosidin (**9**) [63]. Papers concerning the compounds cited above that have been already taken into consideration in other reviews will be mentioned here only when it is necessary for the purpose of comparing their bioactivity with that of the other compounds. 

## 2. Radical Scavenging Activity

The occurrence of a strict relationship between the level of cellular oxidative stress and the tissue inflammation status is well known, so it is very important to evaluate the antioxidant potential of any putative anti-inflammatory compound. The comparison of the radical scavenging activity measured both by the ORAC and CUPRAC assays (Table 4) clearly shows that the presence of the −OH at C-5 implies a significant increase in the reduction power of the molecule. 

Oxygenated radical species (ROS) are mainly responsible for the chemical degradation of important molecular cellular components, such as chromatin, and their control is crucial. Moreover, the presence of –OH groups at C-5 and C-7 in the A ring in the flavone framework was found to be essential in the inhibition of ROS generation in rabbit neutrophils activated with serum-opsonized zymosan (OZ) and insoluble ICs (ICIgG) [73]. This study included two flavones from *Citrus* lacking the above-mentioned structural feature, e.g., compounds **33** and **43**, which showed a moderate inhibitory activity (ca. 30 and 60%, respectively, for OZ, and 13 and 45%, respectively, for ICIgG). 

The involvement of tartrate-resistant acid phosphatase (TRAP) in the intracellular generation of reactive oxygen species (ROS) is well known. Table 4 reports the inhibition activity of several PMFs and HPMFs toward this protein.

## 3. Inhibition of Enzymatic Activity

PMFs and HPMFs can be active toward the proteins involved in the very complex multi-pathway activation of tissue inflammation. Prostaglandins are well recognized mediators of the inflammatory process and the inhibition of the key leukotrienes cycloxygenation step involved in their biosynthesis is a very important goal. Some relevant data concerning the reduction of the production of prostaglandins are reported in Table 5. Many other cases are cited within the following discussion.

Some authors reported computational data concerning the relationship between the structure of different known nutraceuticals, including some PMFs and HPMFs, and the inhibition activity on cycloxygenase-2 (COX-2) determined by a docking procedure. Indeed, the statistical parameters obtained, in particular the correlation coefficient R^2^ (0.462 for AutoDock Vina affinities and 0.238 for Gold scores) between the predicted and the experimental values are quite low, even though the authors claimed the opposite [77]. Experimental determinations of COX-2 inhibition are available for different PMFs and HPMFs from *Citrus* taxa (Table 6). 

The inhibiting power toward this enzyme was evaluated for some natural occurring and semisynthetic flavones [76] and some relevant structural features that can enhance the bioactivity include the presence of a methoxy group at C-8 and of free phenolic –OH at C-5 and C-7. The type of substitution in ring C seems to be less important. Hence, whereas compounds **18** and **26** were inactive, compound **46** was found to be moderately active and **65** was significantly active (Table 6). Other authors investigated the structure–activity relationship for the COX-2 inhibition for a variable number of PMFs and HPMFs [52] by 2D-QSAR methods and obtained a significant correlation coefficient between the predicted versus the experimental mRNA inhibition (*R*^2^ = 0.80). The importance of the presence of the C-2, C-3 double bond as well as the methoxy group at C-4′ was highlighted.

Interestingly, the presence of the above-mentioned alkenyl moiety was also recognized as advantageous in the β-glucuronidase release stimulated by f-MetLeuPhe in human neutrophils [85]. On the other hand, unlike for the case of COX-2, the glucuronidase release appears to be favored by the presence of a phenolic −OH at C-4′, even though it is not very clear from the data reported in this work, whether the main structural contribution to this bioactivity has to be attributed to the 4′ −OH or perhaps to the presence of –OH at C-5 and C-7. However, all the tested PMFs lacking any −OH were inactive (compounds **25**, **44**, **45**, **63**, and **71**).

Several other enzymes involved in several biochemical pathways can be considered as valuable targets to control the inflammation status and both PMFs and HPMFs were proven to be active (Table 6). Compounds **4**–**6** and **14** were also proven to be ineffective as collagenase inhibitors [86], unlike other flavonoids not present in the *Citrus* genus. 

## 4. Inhibition of Nitric Oxide (NO) Production and the Related Biochemical Effects In Vitro

Nitric oxide (NO) is involved in a number of important physiological mechanisms and its level can be raised up as a consequence of an overexpression of the inducible nitric oxide synthase (iNOS), the isoform of NOS produced as a response to the stimulus by pro-inflammatory cytokines. The expression of iNOS can be activated by the translocation of the κB nuclear factor (NF-κB) to the nucleus. It is worth noting that a number of PMFs and HPMFs are able to reduce the levels of both NO (Table 7) and iNOS mRNA, as well as regulate the activity of NF-κB, as described below.

Table 7 reports data taken from several references and, where possible, they are converted in homogeneous units for the purpose of a suitable comparison. Sometimes different values were obtained by different authors; these discrepancies may be due to different degrees of purity of the isolated compounds, as well as by the use of completely different determination methods. However, it is our opinion that the data reported can be considered on the whole to be quite coherent.

Compounds **5**, **14**, and **27** were able to inhibit NO production in LPS-activated RAW267.4 cells, showing remarkable IC_50_ values (Table 7) [83]. Two structural features shared by these compounds are the lack of both the −OH at C-5 and the −OMe at C-3. The three compounds were demonstrated to act at the transcriptional level by inhibiting the expression of the inducible NO Syntase (iNOS) mRNA. Compound **5** was significantly capable to limit the expressions of iNOS, p-IκBα and p-NF-κB in human HaCaT cells stimulated by propionibacterium acnes, thus confirming its potential as an anti-acne agent [107]. The same molecule, when administrated to rats at doses of 100 mg/Kg bw, was able to renormalize the NED induced overexpression of pro-inflammatory proteins (iNOS, COX-2) and NF-κB in HCC cells obtained from pre-neoplastic nodules [108].

Compound **27** showed also a strong effect in inhibiting NO and IL-6 production, both in Raw267.4 and in GES-1 cells. The effect being relevant at only 3 μM [102]; a similar activity was disclosed for compound **52**. Further, a mild effect in the TNFα release was proven for the three compounds (IC_50_ ca. 300 μM). On the contrary, compound **27** displayed a strong reduction effect toward TNF-α release in human mast cells [109]. Furthermore, **27** was found to be effective in preventing inflammatory reactions in human mast cells stimulated by neuropeptides substance P (SP) and neurotensin (NT) [110]. This compound inhibited the mTOR-mediated production of TNF, CXCL8, and VEGF and downregulated the gene expression of these proteins. It also inhibited the degranulation of MCs. The beneficial effect of **27** was still confirmed in a study where the inflammatory reaction of human MCs was induced by substance P together with IL-33 [111]. The pre-treatment with **27** 100 μM implied the inhibition of secretion and gene expression of IL-1β, procaspase-1, and pro-IL-1β.

The effect of **27** was higher than that of its non-methoxylated analogue luteolin, even though the very low solubility and oral availability of the highly lipophilic **27** can be an obstacle for its eventual pharmacological development.

A similar effect was reported [106] in the RAW274,5 cell system for the NO production inhibited by the tetramethoxy derivative **25**. This compound showed also a remarkable inhibition on TNFα release and a potent action toward PGE_2_ and IL-6 production that were almost nullified upon treatment at 100 μM. IL-1β was instead reduced by a much lesser extent (ca. 40%) at the same concentration of the tested compound. A similar pattern of inhibition of the production of pro-inflammatory factors was established for compound 25 by other authors [93]. They described the downregulation of gene activity for the expression of iNOS, COX-2, IL-6, and TNF-α when murine LPS-activated Raw267.4 cells were treated with 25 at 50, 100 μM. This bioactivity was related to the modulation of the NF-κB pathway by 25 that was able to reduce the translocation of p50 and p65 subunits by decreasing the phosphorylation of IKKβ.

With the use of the same cellular system, Li et al. [95] found a significant inhibiting power in NO generation for compounds **57** and **58**, compared to that of **63**, which was much lesser (Table 7). This result was in accordance with a downregulation of both iNOS and COX-2 expressions for the two most active compounds.

Similar effects were investigated for compound 58 formed in the phase I metabolism of **63** [112]. This compound dramatically reduced the NO production (Table 7) and the proinflammatory cytokines PGE_2_, IL-1β, and IL-6, acting by the inhibition of the transcription of both COX-2 and iNOS. Furthermore, the anti-inflammatory activity of **58** was also related to the inhibition of translocation of both NF-κB and AP-1, as well as the activation of NRf2 and its dependent genes NQO-1 and OH-1.

An interesting comparison was made between the anti-inflammatory activity of compounds **20** and **36**, structurally differing only for the presence of a phenolic OH in C-4′ of the former versus a methoxy group in the latter [94]. The substitution of −CH_3_ with a -H was responsible for a decrease in some inflammatory parameters in RAW264.7 cells, such as NO (Table 7) and IL-1β production, as a consequence of reduced expression of iNOS and IL-1β mRNA. On the other hand, some others factors remained substantially unchanged, i.e., PGE_2_ and COX-2 mRNA. Finally, the OH-1 mRNA expression was higher after treatment with **20** than with **34** at the same dose of 2 μM. These differences may be justified on the grounds of the difference in bioavailability related to the reduced hydrophilicity of **34**, with respect to **20**.

In another investigation aiming to evaluate the inhibition of TNF-α release in LPS activated RAW264.7 cells, compounds **44**, **63**, and **71** were all weakly active [113].

Compounds **5** and **33** were found to inhibit the expression of the enzymes involved in the inflammatory response of LPS-activated RAW267.4 cells as they significantly reduced the mRNA expression of IL-1β, TNF-α, IL-6, COX-2, and iNOS [114]; iNOS and COX-2 were deeply downregulated in the same cell type by compound **17** at 10 μM [115]. Further, IL-1β and TNF-α were also effectively reduced in LPS-activated renal epithelia HK-2 cells by compounds **3**, **17**, **25**, and **45** [116]. The antiinflammatory activity of 17 was also proven for another biological model, LPS-activated THP-1 monocytic leukemia cells, where this compound inhibited IL-6 release [117]. Sinensetin (**47**) inhibited the release of several cytokines and chemokines, by reducing their mRNA expression (IL-6, IL-8, IP-10, MCP-1, and TNF-α), which are involved in the inflammation process induced by the H1N1 influenza-A virus on A549 cells [118].

Another interesting structural comparison was proposed by During et al. [119], which evaluated the effect of −OH methylation in the capacity to reduce pro-inflammatory factors in intestinal caco-2 cells, in which the inflammatory response was induced by IL-1β. For example, compound **5** was significantly active in limiting the release of IL-6, MCP-1, COX-1 and COX-2, unlike for chrysin, which is the non-methoxylated form of **5**. The same compound was able to partially reduce the amount of IL-8 and the activation of NF-κB.

Furthermore, other PMFs were compared with their related hydroxylated counterparts (**3** vs. 3,4-dihydroxyflavone, **14** vs. apigenin, **27** vs. luteolin, and **43** vs. quercetin) in their inhibiting power toward the production of IL-6, IL-8, MCP-1, and PGE2. The authors were able to summarize the results obtained with a number of structure-activity inferences, i.e., methylation of the 5- and 7-hydroxyl groups on the A-ring increases the bioactivity, as well as the lack of methylation at the 3′-hydroxyl group on the B-ring, and finally the methylation of the 3-hydroxyl group on the C-ring.

In another investigation, comparing quercetin with different derivatives [70], the per-methylation of this compound yielded **43**, and dramatically reduced both the antioxidant and the anti-inflammatory activities measured by several tests: DPPH scavenging (IC_50_ μg/mL 60 vs. 1.3), FRAP (μg ascorbic acid 0 vs. 2), lipids peroxydation (IC_50_ μg/mL 85 vs. 5), TXB2 inhibition (IC_50_ μg/mL 80 vs. 55), PGE_2_ inhibition (IC_50_ μg/mL 58 vs. 15), 12-HHT (IC_50_ μg/mL 50 vs. 22), and 12-HETE (IC_50_ μg/mL 79 vs. 5). It is worthy to note how the inhibition in the transcription of some inflammatory factors in SW1353 cells was dependent upon the number of methoxy substituents in the flavone core [120]. The order of inhibition power toward the mRNA expression of IL-1β and IL-6 was pentamethoxyflavone **43** > trimethoxyflavone **14** > dimethoxyflavone **5**. On the other hand, the effect on the mRNA expression of TNF-α was less differentiated. 

Compound **45** was also found to be active in inhibiting the superoxide anion production in neutrophils activated with the chemotactic peptide *N*-formylmethionine-leucyl-phenylalanine (FMLP). This activity was associated with a remarkable reduction of the elastase release [121]. Other authors were able to show that the presence of the phenolic −OH at 4′ strongly increased the inhibiting power toward the NO production in LPS activated RAW 264.7 macrophages [105]. Indeed, compound **38** was the most active PMF with respect to the NO inhibition (Table 7). Furthermore, this compound at 40 mM completely suppressed the PGE_2_ production in this cell model. 

Similar dose-depending effects on RAW 264.7 cells were envisaged for compound **67** that effectively reduced the production of NO (Table 7) and PGE_2_ (Table 5) [76]. Other pro-inflammatory factors, such as TNF-α and IL-1β, were restored to physiological levels in the presence of **67** al 40 μM. These effects were associated with the inhibition of NF-κB translocation and activation. Compound **67** was also investigated for its effect in the significant inhibition of the TPA-induced mouse skin inflammation [122] involved in skin tumor genesis. The mRNA expressions of both iNOS and COX-2 were reduced significantly (5-fold and 6-fold respectively) after the topical application of a solution 5-15 mM in acetone for **67**. These effects were accompanied, also in this case, by the inhibition of NF-κB activity and the inhibition of translocation of this protein due to the stoppage of the phosphorylation of its inhibitor IkBα.

The relevant inhibition of the generation of inflammatory proteins on a transcriptional level on SW982 cells stressed with inflammatory cytokines was related to the number of methoxy groups in the flavone framework [123]. The mRNA expression of IL-6, IL-1β, and COX-2 followed the order pentamethoxyflavone **43**, which was significantly higher than trimethoxyflavone **14**, and slightly higher than dimethoxyflavone **5**. The range of mRNA expression observed was once to twice with respect to the positive control (cells untreated with cytokines) for **5** and **14**, whereas **43** was poorly active (mRNA expression 25 to 70 times higher). These results could be explained by the lower polarity, which means lower bioavailability because of the presence of a higher number of methoxy substituents. On the other side, the expression of TNF-α mRNA was much lesser dependent on the structure of the three compounds.

Gardenin A (**68**), a hexamethoxyflavone bearing a hydroxyl group at C-5, was shown to be a potent anti-inflammatory agent in PMA/ionomycin-induced EL-4 cells. This compound reduced IL-5 and ROS production and induced HO-1cexpression through the transcription factor PPARγ. Overall, this data accounts for a possible application of HPMF in controlling the oxidative stress associated with asthma [124].

Compounds **34**, **35**, and **52** were investigated for their anti-inflammatory potential by employing carrageenan-challenged peripheral blood mononuclear cells (PBMCs) as a biological model [125]. The oxidative status of the lysate cells was evaluated by determining the levels of MDA, GSH, SOD, and CAT. Whereas the pre-treatment with the three compounds significantly mitigated MDA accumulation and SOD reduction associated with the inflammation status, GSH and CAT remained unaffected. Furthermore, the levels of inflammation markers COX-2, iNOS, MCP-1, IL-1β, IL-6, and MPO were notably decreased in cells treated with compounds **1** and **2** at 10 mM, whereas compound **3** was not effective in modulating the ratio of COX-2, iNOS, and MCP-1.

The inflammatory pathway in rat basophilic leukemia (RBL-2H3) cells was inhibited on a transcriptional level by both compound **5** and **33**, which reduced both the protein concentration of TNF-α, IL-4, and MCP-1, and the expression of their mRNA [126]. The effect of these compounds was higher than that of the known anti-inflammatory molecule nobiletin (**65**). The cells were more responsive when sensitized by a degranulation- inducing antigen rather than with a calcium ionophore. The same biological target was involved in a comparative study concerning compounds **63** together with its 3-hydroxyderivative, natsudaidain (**66**) [127]. Both compounds had a fable effect on the histamine release and they both inhibited the expression of COX-2 and TNF-α to a significant extent. The presence of an extra –OH group in **68** had a slightly beneficial effect.

## 5. Anti-Neuroinflammatory Activity of *Citrus* PMFs and HPMFs

The role of microglia in neurodegeneration is an expanding area of biomedical investigation that requires the involvement of suitable biological models, such as immortalized BV-2 cells that have been being employed in many studies in recent years. These macrophage-like cells are significantly present in CNS and are involved in some significant neurodegenerative pathologies such as Alzheimer’s, Huntington’s, and Parkinson’s diseases.

The influence of HPMF **67** in the NO production and regulation mechanism was investigated in microglia BV2 cells [98]. The relevant inhibition in the NO production in LPS-activated BV2 cells, treated with **67**, was accompanied by a downregulation of iNOS expression and by the inhibition of NF-κB activity and nuclear translocation. Furthermore, the reduction of NO in these cells was also associated with an overexpression of heme oxygenase 1 (OH-1) enzyme induced by compound **67** and stimulated by the activation of the nuclear factor Nfr2. Interestingly, the activation of the nuclear factor by **67** was reversed by the knockdown of its activator gene obtained by a specific siRNA.

The protective effect toward the neuroinflammation process in BV2 microglia was also investigated for HPMFs **9** [97] that displayed a remarkable IC_50_ value for the NO inhibition (27 μM). In the same work, the inhibitory effect of compound **9** on NO production was evidenced for other cell types such as HAPI microglial, primary astrocytes, and RAW 254,7. Furthermore, compound **9** significantly reduced the levels of the pro-inflammatory factors IL-1β, COX-2, iNOS, and TNF-α in BV2 cells. On the other hand, compound **33** was ineffective in the inhibition of the production of TNF-α, whereas the same compound was established as an effective inhibitor for the production of PGE_2_ (IC_50_ 16.3 μM) [88,128].

It is worthy to note as even minimal structural differences in the substitution pattern of the flavone framework can cause relevant differences in the mechanism of the anti-inflammatory action. For example, compounds **44**, **52**, and **63** differ only in the presence of one more methoxy group in **63**, and one methyl less in **44** with respect to compound **52**. The three compounds showed a comparable inhibition power for the NO production in BV2 cells and a similar ability in reducing the expression of the cytokines IL-1β, IL-6, and TNF-α [129]. However, whereas both **44** and **52** reduced the expression and the phosphorylation of JAK2, as well as the phosphorylation of STAT3, **62** was ineffective toward the expression and the phosphorylation of STAT3. The anti-inflammatory activity of compounds **52** and **65** in BV2 cells was confirmed in their ability to downregulate the transcription of iNOS and IL-1β mRNA [130]; similar effects were demonstrated for compound **71**. The last compound (**63**) also showed mild activity (IC_50_ ca. 20 μM) in the inhibition of the NF-κB activation [131].

The anti-inflammatory activity of **44** was responsible for a notable neuroprotective effect in ischemia/reperfusion (I/R)-injured rat brains [124,132]. Compound **44** significantly decreased the brain tissue pathological parameters such as water content, brain edema, infarct volume, neurological score, and Evans blue leakage. Some relevant pro-inflammatory factors were also downregulated after administration of 5–20 mg/Kg bw of **44**; these include IL-1β, IL-6, TNF-α, TLR-4, and IFNG-γ.

Compound **45** showed anti-inflammatory protection toward SH-SY5Y cells with amyloid β25-35-induced oxidative stress [133]. The compound was efficient in reducing MDA and increasing the SOD and CAT activities at 20–40 μM. Nitric oxide production, IL-1β, and TNF-α were also inhibited together with iNOS and COX-2 mRNA expressions. Further the translocation of the p65 subunit of NF-κB was downregulated as well as TLR-4 expression.

## 6. Anti-Inflammatory Activity In Vivo of *Citrus* PMFs and HPMFs

Compound **9** has been proven to act efficiently against the cartilage destruction in an osteoarthritis mouse model. The biochemical basis of this in vivo effect was investigated thoroughly in vitro, and it was shown that the mechanism of action of this compound is the inhibition of IκB degradation in NF-κB pathway [134]. The same compound appears to be partially effective in reducing ear edema in mice by 23.2% (1 mg/ear) [135]; this activity was related to a notable reduction of NF-κB activation (77%).

Compound **58** effectively reduced ear edema in mice at a dose from 2 to 4 μmol/ear [96]. This effect was accompanied with a strong downregulation of IL-1β, IL-6, and TNF-α, in addition to a reduction of mRNA expression of COX-2, iNOS, and MMP9. Similar effects were disclosed for compounds **5** and **27** that, at a dose of 0.5 mg per ear, were demonstrated to be effective in reducing the ear edema volume (ca. 53 and 50%, respectively), induced by 12-0-tetradecanoylphorbol acetate (TPA) [136]. The same biological model was applied to test retusine (**35**), which displayed an IC_50_ of 0.43 mg/ear [91]. The same group determined the reduction of the paw edema in mice, induced by either TPA and carrageenan, and the administration at 20 mg/Kg of **35** was able to reduce the edema volume by ca. 50%. 

Compound **5** was also investigated using rat paw edema as a model in vivo, showing a notable volume reduction (from 45 to 67%) when administrated orally at doses from 300 to 1200 mg/Kg bw [137]. The same authors also proved a modest reduction effect on carrageenan-induced pleurisy and on cotton pellet-induced granuloma formation (4.5% inhib. at 300 mg/Kg bw). This compound was able to reduce the PGE_2_ production by about 70%. A different number of flavonoids were compared for their oral anti-inflammatory activity in the rat carrageenan-induced hind paw edema test, to gain information on the structure activity relation [138]. Three of the compounds included in these investigations are flavonoids from *Citrus*, i.e., compounds **5**, **14,** and **43**. These compounds had volume inhibition rates of 43.7, 19.5, and 25.3%, respectively, at a dose of 300 mg/Kg bw. The authors concluded that the presence of the pyrene ring and the methoxy groups at C-5 and C-7 are essential for this kind of anti-inflammatory activity. 

Compounds **35** and **71** had a similar effect on paw edema (about 30% for both compounds after 7 h) after an intraperitoneal administration [139]. On the other hand, the result in the cotton pellet-induced granuloma test (chronic inflammation) was 16 and 21% inhibition, respectively, higher than that reported for compound **5**.

The relevant action of compound **6** against acute paw inflammation in rats was reported. This compound is characterized by the presence of two methoxy groups at C-7 and C-4′ [140]. The dose-dependent effect was maximized at 100 mg/Kg bw (90%). A reduction in the number of inflammation biochemical indexes accompanied the in vivo effect of this molecule, that is the reduction of both COX-1 and COX-2 activities (IC_50_ 52 and 24 μM, respectively), as well as the significant decrease in the production of both TNF-α and IL-1β.

Apparently, a higher number of methoxy groups is favorable to obtain a better bioavailability for this type of chronic inflammation test. This generalization is enforced by the results of other groups [141] that proved a quasi-linear correlation between the paw edema volume reduction and the number of methoxy groups in the compounds given at 75 mg/Kg bw, the most active compound being the pentamethoxyflavone quercetine (**43**).

Inflammation is one of the physiological responses to the allergy reaction in tissues, such as delayed-type hypersensitivity (DTH). Demethylnobiletin (**52**) was recognized to be able to modulate DHT induced in mice with the allergen oxazolone, dinitrofluorobenzene (DNFB), and sheep red blood cells (SRBC) by reducing cell infiltration and by suppressing the release of inflammation mediators [90]. Significant reduction rates were observed for IL-2 (IC_50_ 1.63 μM), IL-4 (IC_50_ 2.67 μM), TNF-α (IC_50_ 0.66 μM), IL-1β (46% inhib. at 2.5 μM), and interferon-γ (IC_50_ 1.35 μM). In the same study, compound **52** was effective in reducing NO production (Table 7), even though iNOS expression remained surprisingly unaffected. 

Furthermore, **52** was able to restore the physiological level of TNF-α in mice when a pathological increase of this factor was induced by benzo[a]pyrene and DSS [142].

The in vivo anti-inflammatory action of **52** was confirmed by investigating the hepatoprotective effect of this compound in BULB/c mice treated with CCl_4_. The remarkable protective action of **52** toward ROS-mediated apoptosis in HepG2 cells was explained with the downregulation of CYP2E1 and hepatic SOD expressions, as well as with the notable reduction of the hepatic levels of MDA and GSH [143].

Compounds **17** and **45** confirmed the anti-inflammatory potential disclosed in vitro (see above) in the in vivo xylene-induced ear edema test. They were able to remarkably reduce the auricle swelling at a dose of 50 mg/Kg [116].

Compound **44** was active in restoring the level of pro-inflammatory factors in mice with dextran sulfate sodium (DSS)-induced colitis [144]. The levels of TNF-α, IL-1β, and IL-10 were significantly and dose-dependently downregulated after dietary administration of **44** (0.04 and 0.08%). On the contrary, the moderate reduction of IL-6 level was not statistically significant. Further, this compound was effective in the control of glucose- induced oxidative stress in mice [145]. It was capable of reducing both the 8-hydroxy-2-deoxy guanosine release, indicative of an oxidative DNA damage, and the ROS generation in diabetic podocytes when administrated at a dose of 10 mg/Kg bw. A relevant inhibitory activity toward ROS production was also disclosed for compound **22** at 10 μM in osteoclast lineage cells. The ROS increase induced by sRANKL was blocked by the simultaneous treatment of cells with sRANKL and **22**. This activity was part of the mechanism by which this compound was able to suppress the inflammatory bone destruction [146]. An inhibitory activity of this compound toward NO (Table 7) and TNF-α (Table 8) was also highlighted in mouse macrophage-Like RAW264.7 cells, which was related to the downregulation of the relative mRNA activity [101].

Jecosidin (**9**) inhibited the hind paw edema induced by carrageenan by a volume of 30% at a dose of 20 mg/Kg bw. This activity was accompanied by a significant reduction of the inflammatory markers TNF-α and IL-1β (ca. 50%); the activity on PGE_2_ was instead weak [147]. The same compound (**9**) was found to be significantly active in inhibiting the ear edema in mice (ID_50_ ca. 0.50 μmol/ear) [148].

Compound **20** had similar activity in vivo, showing a reduction of ca. 7% vs. 34% of the positive control indomethacin [149]. Compound **71** had a significantly higher effect after intraperitoneal administration to rats (100 mg/Kg bw), as it was able to reduce the edema by 56%. Oral administration implied a lower hematic concentration of the active compound that reduced the activity at 21% [150]. This in vivo activity was associated with a relevant serum TNF-α reduction (45%) in the treated animals. On the other hand, the molecular mechanism of the anti-inflammatory action of **20** is still unclear, even though an in vitro inhibition toward TNF-α production (IC_50_: 8 μM in THP-1 cells and 4 μM in B16–F10 cells) was also established for this compound [151].

Compound **63** significantly ameliorated the histopathological score and the gastric mucosa injury index in mice suffering from acute gastric lesions induced by ethanol [152]. This effect was accompanied by a significant, linear dose-dependent reduction of several inflammatory markers such as MPO, SOD, GSH, and MDA, as well as serum and the tissues TNF-α and IL-6. PGE_2_ linearly increased within the same dose range of **63**. The same compound also prevented colon carcinogenesis in mice through an anti-inflammatory mechanism on colon cells, where this flavone inhibited the synthesis of IL-1β, IL-6, and TNF-α [96,112,153].

The relative efficacy of PMFs and HPMFs in vivo obviously depends upon their metabolism, bioavailability, and pharmacokinetics after administration. The available literature data concerning this issue are still limited; however, a few useful investigations have been reported for compounds **5** [154], **9** [155,156], and **27** [157,158].

Regardless, because of the narrowness of these studies, it is possible to infer a few general points concerning the kinetics and the metabolism of PMFs that seem to show an average serum cmax of 1–2 μg/mL and a half-life of ca. 3–4 h. The metabolism pathways generally include demethylation at ring A (and eventually C) followed by solfonylation or glycoconiugation at various –OH. Hydroxylation of ring C was observed in compound **5**. Interestingly, the high lipophilicity of compound **71** makes it easy to permeate the blood–brain barrier and exert its neuroprotective effects directly within the CNS after oral or intraperitoneal administration to rats [159]. 

## 7. Spectroscopical Data of Polymethoxy-Flavones, -Flavanones, and -Chalcones Isolated in *Citrus* Genus

**Figure 2:**
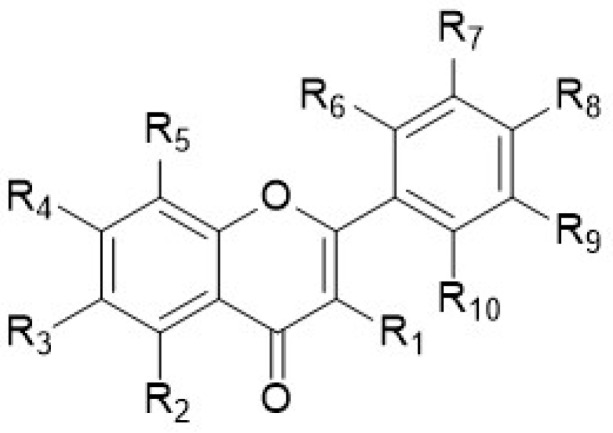
Chemical structure of polymethoxyflavones.

**Figure 3:**
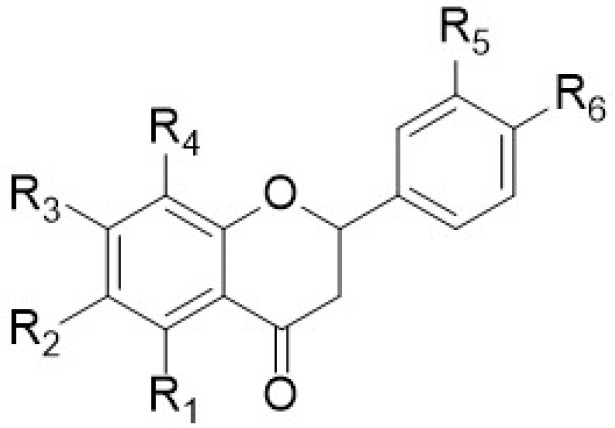
Chemical structure of polymethoxyflavanones.

**Figure 4:**
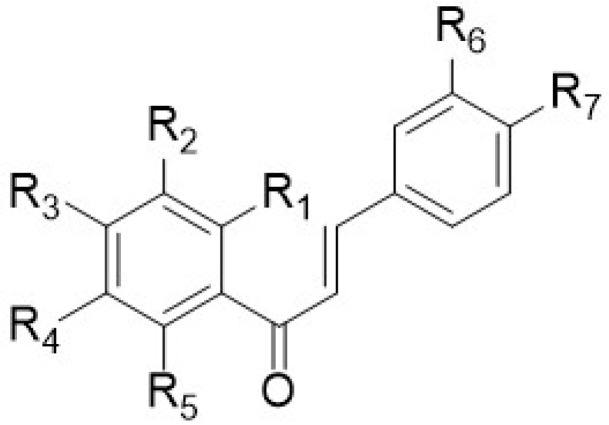
Chemical structure of polymethoxychalcones.

This review, in addition to reporting the data on the anti-inflammatory activity of PMFs and HPMFs extracted from different species of the *Citrus* genus, reports the spectroscopic data of the single isolated polymethoxy-flavones (Figure 2), -flavanones (Figure 3), and -chalcones (Figure 4). This is because, as described in the previous paragraphs, the position of the different functionalities (−OMe or/and −OH) within the flavone skeleton is relevant and basic in the investigated activity.

In particular, the chemical structures of polymethoxyflavones (Table 9) and the spectroscopic values (expressed in ppm) of the single carbon signals (Table 10) and of the related methoxy groups (Table 11) of the polymethoxyflavones (**1**–**71**), and the chemical structures of polymethoxyflavonones (**72**–**77**) and polymethoxychalcones (**78**–**79**) and their ^1^H- and ^13^C-NMR data (Table 12, Table 13, Table 14 and Table 15) are reported. For the identification, an extensive bibliographic search was carried out including only the compounds purely isolated from the different species of the *Citrus* genus. Reported compounds include polymethoxyflavones (71 compounds), polymethoxyflavanones (6 compounds), and chalcones (2 compounds).

Table 9, Table 12 and Table 14 contain all flavonoids with their semisystematic names (and common names) sorted by increasing structural complexity.

All spectroscopic data were obtained using results from articles reporting single isolated polymethoxyflavonoids from the *Citrus* genus, with the help of electronic databases such as SciFinder, Scopus, Google Scholarm, and Web of Science, considering scientific works published until 2022.

## 8. Spectroscopical Data of Glycosylated Flavonoids Isolated in *Citrus* Genus

**Figure 5:**
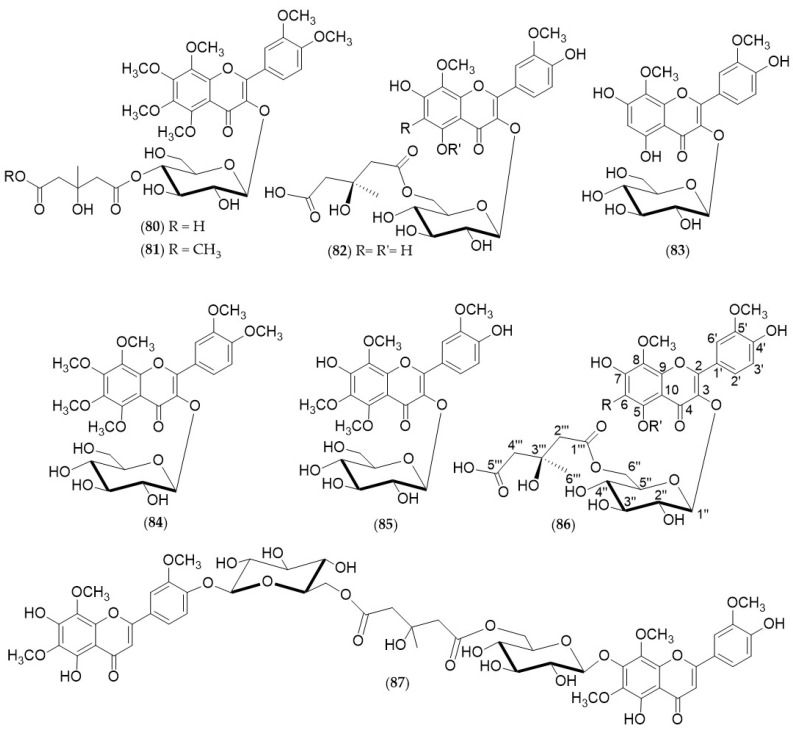

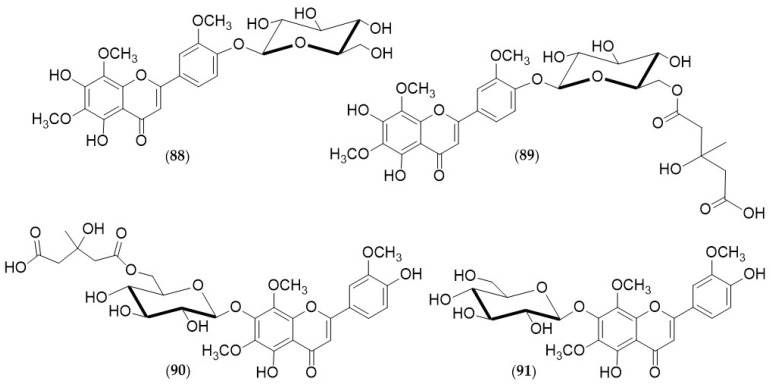
Glycosylated flavones (**80***–***91**) from *Citrus* trees.

Only a few methoxylated and glycosylated flavonoids (**80**–**91**) (Figure 5) have been found and identified mainly from the peels of various *Citrus* trees. They are essentially flavones, characterized by the constant presence of −OMe functionality in position 8 and 5′, glycosylated through β-glycosidic bonds in position 3, or in 7 or in 4′. These compounds were always characterized by the presence of a simple glucose moiety, at most esterified by the 3-hydroxy-3-methylglutaric acid chain (Table 16, Table 17 and Table 18).

## 9. Conclusions

Many species of *Citrus* taxa are part of the daily ordinary diet in several countries and have also been used in traditional medicine because of their beneficial effects on health. In recent years, some of the chemo-preventive and therapeutic effects of *Citrus* were proven by rigorous scientific investigations. The prevention and reduction of inflammation is a truly relevant bioactivity exerted by the *Citrus* secondary metabolites, which is the basis for the prevention of important disorders such as metabolic syndrome, cancer, cardiovascular, and neurodegenerative pathologies. The molecular basis of this anti-inflammatory action is rather complex and involves the regulatory action of *Citrus* PMFs and HPMFs, both at post-transductional and at post-transcriptional levels, as it was described in this paper. However, most of the investigations are performed in vitro or in animal models, such as the murine model that is now considered to poorly mimic human inflammatory diseases [110,190]. 

Studies of the bioactivities on humans are limited and they normally concern epidemiological issues and the use of fruit parts or extracts, rather than the pure isolated compounds. Relevant examples of these s kinds of investigations include the prevention of the metabolic syndrome development and the protection against heart failure by the bergamot extract [191,192] and the antioxidant effect of red orange extract in elderly subjects, which ameliorated their wellbeing (tested by SF-36 QoL and the MRS questionnaires) by reducing the TNF-*α* level and increasing GSH/GSSG ratio after an 8-week intake [193]. Even though these kinds of extracts present a rather complex chemical composition, it is strongly presumable that PMFs and HPMFs can play an important role. Pure compounds were much less investigated clinically, except for a few molecules. For example, nobiletin (**63**) and tangeretin (**44**) were the object of clinical trials aimed to ascertain their beneficial effect on human diseases such as Rhinoconjunctivitis, cognitive disfunctions, and nocturia [194]. It is generally assumed that *Citrus* extracts and their main components are safe and non-toxic and that they are considered as a possible valuable eventual alternative to standard anti-inflammatory drugs such as NSAIDs. However, the acute oral toxicity of sinensitin (**45**) was reported [195]. Hence, deep attention must be paid in order to avoid unsuitable generalizations on presumed healthy effects of *Citrus* extracts and preparations containing PMFs and HPMFs based on scientifically uncontrolled claims. Moreover, there is no doubt that the way is open toward the recognition of PMFs and HPMFs as valuable nutraceuticals or even pharmaceutical leads, likewise for their more established polyphenolic precursor. However, more investigations are necessary in order to better assess the efficacy, the toxicity, and the mechanism of actions of many PMFs and HPMFs and to improve their bioavailability by means of available and suitable drug delivery systems. 

## Figures and Tables

**Table 4:** Radical scavenging activity of PMFs and HPMFs from *Citrus* genus.

Compound	Type of Test	Result (μM)	References
**30**	DPPH (% inhib.)	24.27 at 1000 μM	[64]
**35**		11.44 at 25 μM	[65]
**26, 44, 45, 48, 52, 63, 71**		2–6 at 25 μM	
**20**		3.5 at 100 μM	[66]
**9**		20 at 100 μM	[67]
**10**		25 at 300 μM	[68]
**34**		0 at 300 μM	
**59**		10 at 500 μM	[69]
**43**	DPPH (IC_50_ mg/mL)	0.06	[70]
**67**		0.736	[51]
**52**		0.752	
**63**		3.56	
**9**		1.15	[68]
**9**	Inhibition % of lipoperoxidation in erythrocytes	71 at 300 μM	[68]
**10**		64 at 300 μM	
**34**		28 at 300 μM	
**5**	Reducing capacity Copper(I) ions μM (CUPRAC)	0.08 at 10 μM	[71]
**12**		0.29 at 1 μM	
**14**		0.17 at 10 μM	
**33**		1.05 at 10 μM	
**43**		0 at 10 μM	
**5**	Peroxyl radical-scavenging capacity (ORAC) µM of Trolox equivalent	1.26 at 10 μM	
**12**		1.11 at 10 μM	
**14**		1.51 at 10 μM	
**33**		2.66 at 10 μM	
**43**		0.79 at 10 μM	
**10**	Superoxide anion scavenging (%)	60 at 150 μM	[68]
**34**		29 at 150 μM	
**31**	Superoxide anion scavenging (IC_50_)	>100 μM	[72]
**50**		28.8 μM	
**9**	% LDL-oxidation inhibition (TBARS)	65 at 20 μM	[67]

**Table 5:** Inhibition of PGE_2_ production.

Compound	Test Type	Result	References
**5**	IC_50_ (μM) on RAW264.7 LPS-activated cells	7.43	[74]
**27**		3.13	
**9**	PGE_2_ production in RAW264.7 LPS-activated cells	290 pg/mL at 20 μM	[75]
**67**		1500 pg/mL at 40 μM	[76]
**43**	IC_50_ (μM) on human platelets	200	[70]

**Table 6:** Enzymatic activity of PMFs and HPMFs.

Compound	Type of Test	Result	References
**6**	COX-1 inhibition (%)	63 at 350 μM	[78]
**9**		35 at 10 μM	[79]
**6**	COX-2 inhibition (%)	30 at 350 μM	[78]
**46**		12 at 10 μM	[80]
**65**		65 at 10 μM	
**5**	TRAP activity in RANKL-induced osteoclastic RAW 264.7 cells expressed as % respect to control (Trolox)	200	[71]
**12**		290	
**14**		240	
**33**		400	
**43**		200	
**10**	Xanthine oxidase activity inhibition (%)	28 at 150 μM	[68]
**34**		0 at 150 μM	
**52**	Lipoxygenase activity inhibition (%)	69.0 at 10 μM	[81]
**33** **43**	Phosphodiesterase 1 (IC_50_, μM)	>100	[82]
**33** **43**	Phosphodiesterase 2 (IC_50_, μM)	9.2	
**33** **43**	Phosphodiesterase 3 (IC_50_, μM)	>100	
**33** **43**	Phosphodiesterase 4 (IC_50_, μM)	7.8	
**33** **43**	Phosphodiesterase 5 (IC_50_, μM)	22.8	
**14**	AKT1 (PKB) inhib. % with ATP 75 μM	7 at 10 μM	[83]
	BTK inhib. % with ATP 36 μM	7 at 10 μM	
	CHUK (IKKα). Inhib. % with ATP 9 μM	15 at 10 μM	
	IGF1R Inhib. % with ATP 9 μM	7 at 10 μM	
	IKBKB (IKKβ) Inhib. % with ATP 5 μM	11 at 100 μM	
	IKBKE (IKKe) Inhib. % with ATP 16 μM	2 at 10 μM	
	IRAK4 Inhib. % with ATP 34 μM	3 at 10 μM	
	JAK1 Inhib. % with ATP 87 μM	−10 at 10 μM	
	JAK2 Inhib. % with ATP 31 μM	−7 at 10 μM	
	MAP4K5 (KHS1) Inhib. % with ATP 55 μM	5 at 10 μM	
	MAPK1 (ERK2) Inhib. % with ATP 100 μM	−5 at 100 μM	
	MAPK3 (ERK1) Inhib. % with ATP 45 μM	3 at 10 μM	
	MAPK8 (JNK1) Inhib. % with ATP 100 μM	−5 at 10 μM	
	MAPK9 (JNK2) Inhib. % with ATP 100 μM	5 at 10 μM	
	MAPK10 (JNK3) Inhib. % with ATP 100 μM	3 at 10 μM	
	MAPKAPK2 Inhib. % with ATP 3 μM	2 at 10 μM	
	NEK1 Inhib. % with ATP 119 μM	5 at 10 μM	
	NEK2 Inhib. % with ATP 150 μM	1 at 10 μM	
	PDK 1 Direct Inhib. % with ATP 27 μM	7 at 10 μM	
	PRKACA (PKA) Inhib. % with ATP 4 μM	1 at 10 μM	
	SYK Inhib. % with ATP 25 μM	35 at 10 μM	
	TBK1 Inhib. % with ATP 31 μM	17 at 10 μM	
	ZAP70 Inhib. % with ATP 2 μM	3 at 10 μM	
**3**	NAD(P)H:quinoneoxidoreductase 1 (NQO1): = Concentration required to double the specific activity (μM)	18.0	[84]
**4**		0.085	
**5**		0.2	
**27**		12.5	
**4**	IC_50_ inhib. of inducible NO-syntase on RAW264.7 LPS-activated cells (μM)	5	[84]
**5**		2.5	

**Table 7:** Inhibition of NO production.

Compound	Test Type	Result	References
**5**	IC_50_ (μM) on RAW264.7 LPS-activated cells	33.2	[74]
		5.1	[83]
**9**		14.3	[87]
**12**		88.5	[88]
		60	[83]
**14**		4.6	
**27**		8.7	
		32.5	[74]
**33**		>100	[89]
		16.1	[88]
		66	[83]
**43**		96	
**52**		7.53	[90]
**33**	% Inhibition on murine Mϕ LPS-activated	14.2 at 100 μM	[91]
**41**		8.96 at 50 μM	[92]
**41**	Nitrite production (μM) in Raw 246.7 LPS-activated cells	19 at 50 μM	[92]
**25**		3.5 at 50 μM	[93]
**20**		3 at 30 μM	[94]
**34**		11 at 2 μM	
**57**		27 at 30 μM	[95]
**58**		9 at 30 μM	
		<2 at 30 μM	[96]
**63**		45 at 30 μM	[95]
**67**		8 at 40 μM	[76]
**9**		19 at 20 μM	[67]
		21 at 20 μM	[75]
		6 at 10 μM	[79]
		4 at 50 μM	[97]
**9**	Nitrite production (μM) in primary microglia	1 at 50 μM	[97]
	Nitrite production (μM) in HAPI microglial cells	3 at 50 μM	
	Nitrite production (μM) in primary astrocytes	20 at 50 μM	
**9**	Nitrite production (μM) in BV2 microglia	12 at 30 μM	[97]
**67**		5 at 30 μM	[98]
**1**	% Inhibition on RAW264.7 LPS-activated cells	23 at 50 μM	[99]
**2**		0 at 50 μM	
**74**		40 at 50 μM	
		49 at 200 μM	[100]
**9**		72.5 at 50 μM	[87]
**11**		33 at 50 μM	[99]
**13**		32 at50 μM	
**22**		50 at 30 μM	[101]
**27**		70 at 3 μM	[102]
**35**		90 at 30 μM	[103]
**44**		73 at 200 μM	[100]
		42 at 50 μM	[104]
**45**		75 at 200 μM	[100]
**52**		48 at 200 μM	
		55 at 3 μM	[102]
		40 at 50 μM	[104]
**55**		27.9 at 50 μM	
**63**		47 at 200 μM	[100]
		42 at 50 μM	[104]
**71**		49 at 200 μM	[100]
**38**		95 at 50 μM	[105]
**25**		50 at 50 μM	[106]

**Table 8:** Effect of TNF-α release by PMFs and HPMFs.

Compound	Test Type	Result *	References
**14**	IC_50_ (μM) on RAW264.7 LPS-activated cells	206	[83]
**27**		292	
**25**	% production in RAW264.7 LPS-activated cells	65 at 100 μM	[106]
**22**		48 at 30 μM	[101]

* Data not cited within in the text.

**Table 9:** Chemical structure of polymethoxyflavones (**1**–**71**) isolated in *Citrus* genus.

No.	Name	R_1_	R_2_	R_3_	R_4_	R_5_	R_6_	R_7_	R_8_	R_9_	R_10_
**1**	2′,3′-Dimethoxyflavone	H	H	H	H	H	OMe	OMe	H	H	H
**2**	2′,4′-Dimethoxyflavone	H	H	H	H	H	OMe	H	OMe	H	H
**3**	3′,4′-Dimethoxyflavone	H	H	H	H	H	H	OMe	OMe	H	H
**4**	5,4′-Dimethoxyflavone	H	OMe	H	H	H	H	H	OMe	H	H
**5**	5,7-Dimethoxyflavone (Chrysin dimethyl ether)	H	OMe	H	OMe	H	H	H	H	H	H
**6**	7,4′-Dimethoxyflavone	H	H	H	OMe	H	H	H	OMe	H	H
**7**	5,6-Dihydroxy-7,4′-dimethoxyflavone (Ladanein)	H	OH	OH	OMe	H	H	H	OMe	H	H
**8**	3,5,6-Trihydroxy-7,4′-dimethoxyflavone	OH	OH	OH	OMe	H	H	H	OMe	H	H
**9**	5,7,4′-Trihydroxy-6,3′-dimethoxyflavone (Jaceosidin)	H	OH	OMe	OH	H	H	OMe	OH	H	H
**10**	5,7,4′-Trihydroxy-6,8-dimethoxyflavone (Demethoxysudachitin)	H	OH	OMe	OH	OMe	H	H	OMe	H	H
**11**	2′,3′,4′-Trimethoxyflavone	H	H	H	H	H	OMe	OMe	OMe	H	H
**12**	3,5,7-Trimethoxyflavone (Galangin trimethyl ether)	OMe	OMe	H	OMe	H	H	H	H	H	H
**13**	3′,4′,5′-Trimethoxyflavone	H	H	H	H	H	H	OMe	OMe	OMe	H
**14**	5,7,4′-Trimethoxyflavone (Apigenin trimethyl ether)	H	OMe	H	OMe	H	H	H	OMe	H	H
**15**	5,3′,4′-Trimethoxyflavone	H	OMe	H	H	H	H	OMe	OMe	H	H
**16**	6,2′,3′-Trimethoxyflavone	H	H	OMe	H	H	OMe	OMe	H	H	H
**17**	5-Hydroxy-6,7,4′-trimethoxyflavone (Salvigenin)	H	OH	OMe	OMe	H	H	H	OMe	H	H
**18**	5-Hydroxy-7,8,4′-trimethoxyflavone (Isoscutellarein 7,8,4′-trimethyl ether)	H	OH	H	OMe	OMe	H	H	OMe	H	H
**19**	8-Hydroxy-5,7,4′-trimethoxyflavone	H	OMe	H	OMe	OH	H	H	OMe	H	H
**20**	5,4′-Dihydroxy-6,7,8-trimethoxyflavone (Xanthomicrol)	H	OH	OMe	OMe	OMe	H	H	OH	H	H
**21**	3,5,6-Trihydroxy-7,3′,4′-trimethoxyflavone	OH	OH	OH	OMe	H	H	OMe	OMe	H	H
**22**	5,7,4′-Trihydroxy-6,8,3′- trimethoxyflavone	H	OH	OMe	OH	OMe	H	OMe	OMe	H	H
**23**	3,5,7,4′-Tetrahydroxy-6,8,3′-trimethoxyflavone (Limocitrol)	OH	OH	OMe	OH	OMe	H	OMe	OH	H	H
**24**	3,6,7,4′-Tetramethoxyflavone	OMe	H	OMe	OMe	H	H	H	OMe	H	H
**25**	5,6,7,4′-Tetramethoxyflavone (Scutellarein tetramethyl ether)	H	OMe	OMe	OMe	H	H	H	OMe	H	H
**26**	5,7,8,4′-Tetramethoxyflavone (6-Demethoxytangeretin)	H	OMe	H	OMe	OMe	H	H	OMe	H	H
**27**	5,7,3′,4′-Tetramethoxyflavone (Luteolin tetramethyl ether)	H	OMe	H	OMe	H	H	OMe	OMe	H	H
**28**	6,7,8,4′-Tetramethoxyflavone	H	H	OMe	OMe	OMe	H	H	OMe	H	H
**29**	7,3′,4′,5′-Tetramethoxyflavone	H	H	H	OMe	H	H	OMe	OMe	OMe	H
**30**	3-Hydroxy-5,6,7,4′-tetramethoxyflavone (Eupatorin-5-methylether)	OH	OMe	OMe	OMe	H	H	H	OMe	H	H
**31**	5-Hydroxy-3,6,7,4′-tetramethoxyflavone (Penduletin 4′-methyl ether)	OMe	OH	OMe	OMe	H	H	H	OMe	H	H
**32**	5-Hydroxy-3,7,8,4′-tetramethoxyflavone	OMe	OH	H	OMe	OMe	H	H	OMe	H	H
**33**	5-Hydroxy-3,7,3′,4′-tetramethoxyflavone (Retusin)	OMe	OH	H	OMe	H	H	OMe	OMe	H	H
**34**	5-Hydroxy-6,7,8,4′-tetramethoxyflavone (Gardenin B)	H	OH	OMe	OMe	OMe	H	H	OMe	H	H
**35**	5-Hydroxy-6,7,3′,4′-tetramethoxyflavone (5-Desmethylsinensetin)	H	OH	OMe	OMe	H	H	OMe	OMe	H	H
**36**	6-Hydroxy-5,7,8,4′-tetramethoxyflavone	H	OMe	OH	OMe	OMe	H	H	OMe	H	H
**37**	7-Hydroxy-5,6,8,4′-tetramethoxyflavone	H	OMe	OMe	OH	OMe	H	H	OMe	H	H
**38**	4′-Hydroxy-5,6,7,8-tetramethoxyflavone	H	OMe	OMe	OMe	OMe	H	H	OH	H	H
**39**	5,7-Dihydroxy-6,8,3′,4′-tetramethoxyflavone (Hymenoxin)	H	OH	OMe	OH	OMe	H	OMe	OMe	H	H
**40**	5,3′-Dihydroxy-3,7,4′,5′-tetramethoxyflavone	OMe	OH	H	OMe	H	H	OH	OMe	OMe	H
**41**	5,4′-Dihydroxy-6,7,8,3′-tetramethoxyflavone (8-Methoxycirsilineol)	H	OH	OMe	OMe	OMe	H	OMe	OH	H	H
**42**	3,5,6,8,4′-Pentamethoxyflavone	OMe	OMe	OMe	H	OMe	H	H	OMe	H	H
**43**	3,5,7,3′,4′-Pentamethoxyflavone (Quercetin pentamethyl ether)	OMe	OMe	H	OMe	H	H	OMe	OMe	H	H
**44**	5,6,7,8,4′-Pentamethoxyflavone (Tangeretin)	H	OMe	OMe	OMe	OMe	H	H	OMe	H	H
**45**	5,6,7,3′,4′-Pentamethoxyflavone (Sinensitin)	H	OMe	OMe	OMe	H	H	OMe	OMe	H	H
**46**	5,7,8,3′,4′-Pentamethoxyflavone (Isosinensetin)	H	OMe	H	OMe	OMe	H	OMe	OMe	H	H
**47**	5,7,2′,3′,4′-Pentamethoxyflavone	H	OMe	H	OMe	H	OMe	OMe	OMe	H	H
**48**	6,7,8,3′,4′-Pentamethoxyflavone (Demethylnobiletin)	H	H	OMe	OMe	OMe	H	OMe	OMe	H	H
**49**	3-Hydroxy-5,6,7,8,4′-pentamethoxyflavone (3-Demethylnobiletin)	OH	OMe	OMe	OMe	OMe	H	H	OMe	H	H
**50**	5-Hydroxy-3,6,7,8,4′-Pentamethoxyflavone (5-Hydroxyauranetin)	OMe	OH	OMe	OMe	OMe	H	H	OMe	H	H
**51**	5-Hydroxy-3,7,8,3′,4′-pentamethoxyflavone (Gossypetin pentamethylether)	OMe	OH	H	OMe	OMe	H	OMe	OMe	H	H
**52**	5-Hydroxy-6,7,8,3′,4′-pentamethoxyflavone (5-Demethylnobiletin)	H	OH	OMe	OMe	OMe	H	OMe	OMe	OH	H
**53**	5-Hydroxy-6,7,3′,4′,5′-pentamethoxyflavone (Umuhengerin)	H	OH	OMe	OMe	H	H	OMe	OMe	OMe	H
**54**	7-Hydroxy-3,5,6,3′,4′-pentamethoxyflavone	OMe	OMe	OMe	OH	H	H	OMe	OMe	H	H
**55**	7-Hydroxy-5,6,8,3′,4′-pentamethoxyflavone (7-Demethylnobiletin)	H	OMe	OMe	OH	OMe	H	OMe	OMe	H	H
**56**	8-Hydroxy-3,5,6,7,4′-pentamethoxyflavone	OMe	OMe	OMe	OMe	OH	H	H	OMe	H	H
**57**	3′-Hydroxy-5,6,7,8,4′-pentamethoxyflavone (3-Hydroxytangeretin)	H	OMe	OMe	OMe	OMe	H	OH	OMe	H	H
**58**	4′-Hydroxy-5,6,7,8,3′-pentamethoxyflavone (4′-Demethylnobiletin)	H	OMe	OMe	OMe	OMe	H	OMe	OH	H	H
**59**	3,5,6,7,8,4′-Hexamethoxyflavone (3-Methoxytangeretin)	OMe	OMe	OMe	OMe	OMe	H	H	OMe	H	H
**60**	3,5,7,8,2′,5′-Hexamethoxyflavone	OMe	OMe	H	OMe	OMe	OMe	H	H	OMe	H
**61**	3,5,7,8,3′,4′-Hexamethoxyflavone (Gossypetin hexamethyl ether)	OMe	OMe	H	OMe	OMe	H	OMe	OMe	H	H
**62**	3,6,7,8,2′,5′-Hexamethoxyflavone	OMe	H	OMe	OMe	OMe	OMe	H	H	OMe	H
**63**	5,6,7,8,3′,4′-Hexamethoxyflavone (Nobiletin)	H	OMe	OMe	OMe	OMe	H	OMe	OMe	H	H
**64**	5,6,7,3′,4′,5′-Hexamethoxyflavone	H	OMe	OMe	OMe	H	H	OMe	OMe	OMe	H
**65**	5,7,8,3′,4′,5′-Hexamethoxyflavone (Bannamurpanisin)	H	OMe	H	OMe	OMe	H	OMe	OMe	OMe	H
**66**	3-Hydroxy-5,6,7,8,3′,4′-hexamethoxyflavone (Natsudaidain)	OH	OMe	OMe	OMe	OMe	H	OMe	OMe	H	H
**67**	5-Hydroxy-3,6,7,8,3′,4′-hexamethoxyflavone	OMe	OH	OMe	OMe	OMe	H	OMe	OMe	H	H
**68**	5-Hydroxy-6,7,8,3′,4′,5′-hexamethoxyflavone (Gardenin A)	H	OH	OMe	OMe	OMe	H	OMe	OMe	OMe	H
**69**	7-Hydroxy-3,5,6,8,3′,4′-hexamethoxyflavone	OMe	OMe	OMe	OH	OMe	H	OMe	OMe	H	H
**70**	8-Hydroxy-3,5,6,7,3′,4′-hexamethoxyflavone	OMe	OMe	OMe	OMe	OH	H	OMe	OMe	H	H
**71**	3,5,6,7,8,3′,4′-Heptamethoxyflavone	OMe	OMe	OMe	OMe	OMe	H	OMe	OMe	H	H

**Table 10:** ^13^C-NMR of polymethoxyflavones’ (**1**–**71**) skeleton isolated in *Citrus* genus.

No.	C_2_	C_3_	C_4_	C_5_	C_6_	C_7_	C_8_	C_9_	C_10_	C_1′_	C_2′_	C_3′_	C_4′_	C_5′_	C_6′_	Solvent	References
**1**	161.7	-	177.6	125.4	124.5	134.3	118.3	155.9	123.0	111.2	147.1	153.0	111.2	120.5	116.0	DMSO-*d*_6_	[26]
**2**	160.6	110.2	177.1	124.7	125.2	134.1	118.4	155.8	123.1	112.3	159.5	99.1	163.2	106.3	130.4	DMSO-*d*_6_	[160]
**3**	162.7	105.7	177.0	124.7	125.3	134.0	118.5	155.6	123.3	123.3	109.4	149.0	151.9	111.7	119.9	DMSO-*d*_6_	[160]
**4**	160.1	106.9	176.4	159.0	107.2	134.1	109.9	157.5	113.7	122.9	127.8	114.5	161.9	114.5	127.8	DMSO-*d*_6_	[161]
**5**	163.9	109.0	177.4	160.5	92.8	160.8	96.1	159.8	109.3	131.5	125.9	128.8	131.1	128.8	125.9	CDCl_3_	[162]
**6**	162.3	105.2	176.3	126.1	114.5	163.7	100.9	157.4	117.1	123.3	128.0	114.5	162.0	114.5	128.0	DMSO-*d*_6_	[161]
**7**	163.1	102.9	182.0	146.1	129.9	154.2	90.9	149.5	105.0	122.8	127.9	114.3	162.0	114.3	127.9	DMSO-*d*_6_	[163]
**8**	146.4	135.9	176.0	144.9	129.2	154.6	90.7	148.8	104.3	123.3	129.2	113.9	160.4	113.9	129.2	DMSO-*d*_6_	[164]
**9**	163.7	102.7	182.1	152.7	131.3	157.3	94.3	152.4	104.0	121.5	110.1	150.7	148.0	115.7	120.3	DMSO-*d*_6_	[165]
**10**	165.0	103.6	183.7	149.8	132.4	151.3	117.0	146.7	104.6	123.4	129.2	116.5	162.0	116.5	129.2	Acetone-*d*_6_	[166]
**11**	160.9	110.0	176.6	124.4	124.0	133.8	118.0	155.9	122.8	108.0	152.0	141.9	155.6	108.0	118.0	DMSO-*d*_6_	[26]
**12**	152.0	141.4	173.6	160.5	95.5	163.5	92.1	158.4	109.1	130.5	127.7	128.0	129.0	128.0	127.7	CDCl_3_	[167]
**13**	162.0	106.3	176.6	124.9	124.3	133.5	118.0	155.2	123.0	126.0	104.1	152.9	140.8	152.9	104.1	DMSO-*d*_6_	[26]
**14**	160.4	106.8	176.0	160.0	93.3	163.7	96.2	159.3	108.5	123.2	127.6	114.4	161.9	114.4	127.6	DMSO-*d*_6_	[168]
**15**	161.0	106.4	178.3	159.7	111.1	133.6	108.6	158.2	114.5	123.9	110.1	149.2	151.8	108.0	119.7	CDCl_3_	[169]
**16**	161.3	110.4	176.6	104.6	156.6	123.3	120.1	150.6	123.8	126.0	147.1	153.0	115.8	124.5	120.6	DMSO-*d*_6_	[161]
**17**	163.6	103.3	182.3	152.0	131.9	158.7	91.6	152.7	105.1	122.7	128.3	114.6	162.4	114.6	128.3	DMSO-*d*_6_	[34]
**18**	163.9	103.8	182.7	157.5	95.7	158.5	128.9	149.4	104.8	123.6	128.1	114.6	162.7	114.6	128.1	CDCl_3_	[164]
**19**	159.6	105.9	176.2	151.8	94.0	152.0	127.9	146.8	108.0	123.2	127.8	114.3	161.7	114.3	127.8	DMSO-*d*_6_	[164]
**20**	164.1	102.6	182.5	145.5	135.6	152.4	132.6	145.1	106.1	121.0	128.4	116.1	161.4	116.1	128.4	DMSO-*d*_6_	[170]
**21**	146.3	136.1	176.0	144.9	129.2	154.6	90.8	148.7	104.3	123.4	110.7	121.4	150.3	148.3	111.4	DMSO-*d*_6_	[171]
**22**	163.1	102.7	182.0	148.1	131.3	150.5	127.7	145.1	115.7	121.4	109.7	119.9	150.5	115.7	147.8	DMSO-*d*_6_	[164]
**23**	146.5	135.6	176.2	147.2	130.9	150.5	127.6	144.3	102.3	122.1	111.2	148.8	147.3	115.6	121.6	DMSO-*d*_6_	[164]
**24**	160.3	106.1	175.6	97.3	139.8	161.8	157.4	153.9	114.5	123.0	127.8	114.5	151.6	114.5	127.8	DMSO-*d*_6_	[31]
**25**	160.3	106.1	175.6	151.6	139.7	157.4	97.3	153.9	112.0	123.0	127.8	114.5	161.9	114.5	127.8	DMSO-*d*_6_	[161]
**26**	159.4	106.2	175.8	155.6	93.5	156.2	129.8	150.9	107.8	123.1	127.5	114.5	161.7	114.5	127.5	DMSO-*d*_6_	[164]
**27**	160.4	107.5	177.4	160.6	96.0	164.8	92.8	160.0	108.9	123.7	108.3	149.0	151.6	-	119.3	CDCl_3_	[172]
**28**	161.2	107.1	177.2	96.2	157.6	140.3	152.6	154.5	112.9	123.9	127.7	114.4	162.1	114.4	127.7	CDCl_3_	[32]
**29**	162.9	107.3	177.7	127.1	114.4	164.2	100.5	157.9	117.8	127.0	103.7	153.5	141.4	153.5	103.7	CDCl_3_	[173]
**30**	142.6	137.6	171.0	151.0	139.3	157.5	96.9	153.0	110.0	123.5	128.8	114.0	160.1	114.0	128.8	DMSO-*d*_6_	[34]
**31**	155.4	137.9	178.2	151.6	131.6	158.6	91.4	151.8	105.6	122.0	130.0	114.2	161.4	114.2	130.0	DMSO-*d*_6_	[164]
**32**	155.4	137.8	178.3	156.3	95.7	158.2	128.3	147.8	104.5	122.2	129.8	114.3	161.4	114.3	129.8	DMSO-*d*_6_	[164]
**33**	155.5	138.3	178.1	156.4	97.9	165.2	92.5	156.4	105.3	122.1	111.6	148.5	151.4	111.3	122.1	DMSO-*d*_6_	[34]
**34**	163.9	103.4	182.7	148.7	136.0	152.6	132.8	145.4	106.3	122.8	128.4	114.9	162.7	114.9	128.4	DMSO-*d*_6_	[34]
**35**	163.0	104.5	182.5	153.8	133.0	159.0	91.0	155.0	105.5	120.1	112.2	152.4	149.5	110.0	123.9	CDCl_3_	[174]
**36**	160.1	105.7	175.9	141.2	140.7	146.3	137.8	143.6	114.1	123.2	127.6	114.5	161.8	114.5	127.6	DMSO-*d*_6_	[164]
**37**	159.8	105.8	175.7	147.3	139.4	149.1	132.5	147.5	110.6	123.2	127.6	114.6	161.8	114.6	127.6	DMSO-*d*_6_	[164]
**38**	160.7	105.3	175.6	147.4	143.4	150.8	137.7	147.0	114.2	121.3	127.8	115.9	160.7	115.9	127.8	DMSO-*d*_6_	[164]
**39**	163.7	104.0	182.9	148.4	130.9	148.9	127.3	145.8	104.6	123.7	108.8	149.4	152.4	111.3	120.1	CDCl_3_	[175]
**40**	155.3	139.7	178.8	162.0	98.0	165.6	92.2	156.8	106.1	126.0	108.6	149.2	137.8	152.0	105.1	CDCl_3_	[176]
**41**	164.0	102.9	182.5	148.4	135.8	152.4	132.5	145.1	106.1	121.3	110.0	148.0	151.0	115.9	120.3	DMSO-*d*_6_	[170]
**42**	152.5	139.4	172.9	138.4	148.8	103.4	139.9	145.0	118.9	122.6	129.6	114.2	161.0	114.2	129.6	DMSO-*d*_6_	[164]
**43**	152.6	141.2	174.0	158.8	95.8	163.9	92.5	161.1	109.5	123.4	111.3	148.7	150.9	110.8	121.6	CDCl_3_	[177]
**44**	160.4	106.1	175.8	147.6	143.6	162.1	137.8	147.6	114.7	123.1	127.8	114.3	151.0	114.3	127.8	DMSO-*d*_6_	[31]
**45**	159.6	106.7	176.1	151.7	131.8	156.5	94.2	155.8	111.1	123.4	108.9	149.1	151.2	112.3	119.3	DMSO-*d*_6_	[31]
**46**	160.5	107.2	177.9	152.0	92.6	156.3	130.7	156.3	109.1	124.1	108.7	149.3	151.8	111.0	119.7	CDCl_3_	[178]
**47**	160.3	111.5	175.7	158.1	96.1	163.7	93.2	159.8	108.2	117.8	152.1	142.2	155.8	108.2	123.9	DMSO-*d*_6_	[179]
**48**	160.3	106.4	175.7	97.3	139.7	157.4	151.5	153.9	111.7	123.2	109.2	149.0	151.7	112.0	119.4	DMSO-*d*_6_	[31]
**49**	142.9	137.8	171.2	147.0	143.1	150.7	137.4	146.2	112.3	123.5	128.8	114.2	160.3	114.2	128.8	DMSO-*d*_6_	[34]
**50**	155.7	137.8	178.6	148.1	135.4	152.3	132.5	144.4	106.7	122.1	129.9	114.4	161.5	114.4	129.9	DMSO-*d*_6_	[164]
**51**	155.2	138.0	178.3	156.4	95.8	158.2	128.8	147.8	104.5	122.2	111.7	148.4	151.3	110.9	121.9	DMSO-*d*_6_	[34]
**52**	163.5	103.5	182.5	148.5	135.8	152.4	132.5	145.2	106.2	122.6	111.7	148.9	152.3	109.0	119.9	DMSO-*d*_6_	[34]
**53**	163.8	105.4	182.6	153.2	132.9	158.9	90.7	153.1	106.3	126.5	103.9	153.7	141.6	153.7	103.9	CDCl_3_	[180]
**54**	151.8	140.0	171.4	137.4	142.8	158.2	96.1	153.7	109.8	123.8	110.6	148.9	150.5	111.0	120.8	CDCl_3_	[44]
**55**	161.4	106.7	177.3	145.1	140.1	140.3	138.1	145.6	114.2	124.2	108.7	149.4	152.0	111.3	119.7	CDCl_3_	[181]
**56**	143.2	137.9	171.8	147.5	143.5	151.5	137.2	146.9	111.7	123.6	129.0	144.1	160.9	114.1	129.0	CDCl_3_	[45]
**57**	142.8	137.7	171.2	146.9	143.0	150.7	137.4	146.1	112.2	123.5	128.7	114.1	160.2	114.1	128.7	DMSO-*d*_6_	[164]
**58**	161.2	106.6	177.4	148.9	106.6	151.4	138.0	146.9	114.8	123.5	108.2	147.7	148.4	115.0	120.2	CDCl_3_	[182]
**59**	152.5	139.8	172.2	147.2	143.2	150.8	137.4	146.2	114.4	122.5	129.5	114.3	161.0	114.3	129.5	DMSO-*d*_6_	[164]
**60**	152.3	140.9	174.3	92.3	156.3	156.4	152.5	151.0	109.4	123.6	150.9	111.0	121.8	148.7	152.3	CDCl_3_	[32]
**61**	150.8	140.8	174.2	152.2	92.4	156.4	130.4	156.3	109.4	123.6	110.9	148.7	150.9	111.0	121.8	CDCl_3_	[178]
**62**	153.3	140.8	173.7	96.0	157.7	140.2	152.4	153.6	113.1	123.4	151.0	110.9	121.7	148.7	111.4	CDCl_3_	[32]
**63**	160.2	106.3	175.8	143.5	137.6	150.9	147.5	147.1	114.3	123.1	108.9	149.0	151.8	111.9	119.3	DMSO-*d*_6_	[31]
**64**	161.0	108.3	177.2	154.5	140.4	157.8	96.3	152.6	112.9	126.9	103.4	153.6	140.9	153.6	103.4	CDCl_3_	[183]
**65**	160.2	107.8	177.8	151.9	92.3	156.6	130.5	156.3	108.7	126.6	103.1	153.4	140.7	153.4	103.1	CDCl_3_	[183]
**66**	146.9	143.1	172.0	143.6	137.9	150.6	137.5	147.7	111.8	123.9	111.2	149.0	151.7	110.3	121.1	CDCl_3_	[181]
**67**	155.6	138.1	178.7	148.5	135.5	152.5	132.4	144.4	106.8	122.1	110.9	148.1	151.4	111.8	122.0	DMSO-*d*_6_	[34]
**68**	163.6	104.8	183.0	149.5	136.6	153.1	132.9	145.8	107.0	126.3	103.6	153.6	141.5	153.6	103.6	CDCl_3_	[184]
**69**	150.7	142.9	171.8	143.6	147.7	146.9	137.4	146.9	111.7	123.9	110.6	149.0	151.7	111.3	121.1	CDCl_3_	[44]
**70**	142.8	137.8	171.8	147.6	143.5	151.6	137.4	146.8	111.7	123.8	110.3	148.9	150.5	111.1	121.0	CDCl_3_	[45]
**71**	150.9	140.0	172.3	143.1	137.4	151.9	138.9	147.9	114.4	122.5	110.0	148.5	154.1	111.7	121.5	DMSO-*d*_6_	[31]

**Table 11:** ^13^C-NMR of polymethoxyflavones’ (**1**–**71**) methoxy groups occurring in *Citrus* genus.

No.	OMe-3	OMe-5	OMe-6	OMe-7	OMe-8	OMe-2′	OMe-3′	OMe-4′	OMe-5′	OMe-6′	Solvent	References
**1**	-	-	-	-	-	60.2	55.9	-	-	-	DMSO-*d*_6_	[26]
**2**	-	-	-	-	-	56.1	-	55.7	-	-	DMSO-*d*_6_	[160]
**3**	-	-	-	-	-	-	55.8	55.7	-	-	DMSO-*d*_6_	[160]
**4**	-	56.1	-	-	-	-	-	55.5	-	-	DMSO-*d*_6_	[161]
**5**	-	56.5	-	55.8	-	-	-	-	-	-	CDCl_3_	[162]
**6**	-	-	-	55.5	-	-	-	56.0	-	-	DMSO-*d*_6_	[161]
**7**	-	-	-	56.1	-	-	-	55.4	-	-	DMSO-*d*_6_	[163]
**8**	-	-	-	56.2	-	-	-	55.3	-	-	DMSO-*d*_6_	[164]
**9**	-	-	59.9	-	-	-	55.9	-	-	-	DMSO-*d*_6_	[165]
**10**	-	-	60.8	-	61.8	-	-	-	-	-	Acetone-*d*_6_	[166]
**11**	-	-	-	-	-	60.9	60.2	55.8	-	-	DMSO-*d*_6_	[26]
**12**	59.7	55.4	-	56.0	-	-	-	-	-	-	CDCl_3_	[167]
**13**	-	-	-	-	-	-	56.1	59.4	-	56.1	DMSO-*d*_6_	[26]
**14**	-	56.1	-	55.9	-	-	-	55.4	-	-	DMSO-*d*_6_	[168]
**15**	-	56.5	-	-	-	-	56.1	56.1	-	-	CDCl_3_	[169]
**16**	-	-	55.7	-	-	60.6	56.0	-	-	-	DMSO-*d*_6_	[161]
**17**	-	-	60.6	56.5	-	-	-	55.6	-	-	DMSO-*d*_6_	[34]
**18**	-	-	-	56.3	61.7	-	-	55.5	-	-	CDCl_3_	[164]
**19**	-	56.3	-	56.2	-	-	-	55.4	-		DMSO-*d*_6_	[164]
**20**	-	-	60.5	61.8	61.6	-	-	-	-	-	DMSO-*d*_6_	[170]
**21**	-	-	-	56.5	-	-	55.6	55.6	-	-	DMSO-*d*_6_	[171]
**22**	-	-	61.0	-	59.8	-	-	56.0	-	-	DMSO-*d*_6_	[164]
**23**	-	-	-	-	-	-	55.5	-	-	-	DMSO-*d*_6_	[164]
**24**	61.0	-	55.5	61.8	-	-	-	56.4	-	-	DMSO-*d*_6_	[31]
**25**	-	61.8	61.0	56.4	-	-	-	55.5	-	-	DMSO-*d*_6_	[161]
**26**	-	56.3	-	56.1	60.9	-	-	55.4	-	-	DMSO-*d*_6_	[164]
**27**	-	56.0	-	55.7	-	-	55.7	56.2	-	-	CDCl_3_	[172]
**28**	-	-	56.3	61.6	62.2	-	-	55.5	-	-	CDCl_3_	[32]
**29**	-	-	-	55.8	-	-	56.3	61.0	56.3	-	CDCl_3_	[173]
**30**	-	61.9	61.1	56.5	-	-	-	55.3	-	-	DMSO-*d*_6_	[34]
**31**	59.7	-	60.0	56.4	-	-	-	55.4	-	-	DMSO-*d*_6_	[164]
**32**	59.7	-	-	56.5	61.0	-	-	55.4	-	-	DMSO-*d*_6_	[164]
**33**	59.8	-	-	56.2	-	-	55.7	55.7	-	-	DMSO-*d*_6_	[34]
**34**	-	-	60.7	62.0	61.6	-	-	55.7	-	-	DMSO-*d*_6_	[34]
**35**	-	-	61.0	58.5	-	-	56.2	56.1	-	-	CDCl_3_	[174]
**36**	-	61.7	-	61.4	60.8	-	-	55.4	-	-	DMSO-*d*_6_	[164]
**37**	-	61.7	61.1	-	61.1	-	-	55.4	-	-	DMSO-*d*_6_	[164]
**38**	-	61.3	61.4	61.7	61.8	-	-	-	-	-	DMSO-*d*_6_	[164]
**39**	-	-	61.0	-	61.8	-	56.1	56.0	-	-	CDCl_3_	[175]
**40**	60.3	-	-	56.1	-	-	-	61.1	55.8	-	CDCl_3_	[176]
**41**	-	-	60.5	61.8	61.4	-	55.8	-	-	-	DMSO-*d*_6_	[170]
**42**	59.2	56.9	56.7	-	61.0	-	-	55.3	-	-	DMSO-*d*_6_	[164]
**43**	59.9	55.8	-	55.9	-	-	56.1	56.4	-	-	CDCl_3_	[177]
**44**	-	62.0	55.6	61.9	61.5	-	-	61.6	-	-	DMSO-*d*_6_	[31]
**45**	-	61.1	55.8	56.5	-	-	56.4	55.9	-	-	DMSO-*d*_6_	[31]
**46**	-	56.5	-	56.3	61.5	-	56.1	56.0	-	-	CDCl_3_	[178]
**47**	-	56.0	-	56.1	-	61.0	60.5	56.1	-	-	DMSO-*d*_6_	[179]
**48**	-	-	55.7	61.8	55.9	-	61.0	56.4	-	-	DMSO-*d*_6_	[31]
**49**	-	61.8	61.4	61.9	61.5	-	-	55.3	-	-	DMSO-*d*_6_	[34]
**50**	59.7	-	60.5	61.8	61.4	-	-	55.4	-	-	DMSO-*d*_6_	[164]
**51**	59.7	-	-	56.5	61.0	-	55.3	55.6	-	-	DMSO-*d*_6_	[34]
**52**	-	-	60.5	61.7	61.4	-	55.7	55.6	-	-	DMSO-*d*_6_	[34]
**53**	-	-	60.8	56.4	-	-	56.5	60.8	56.5	-	CDCl_3_	[180]
**54**	62.3	61.6	56.4	-	-	-	56.1	56.0	-	-	CDCl_3_	[44]
**55**	-	62.8	62.1	-	61.5	-	56.5	56.0	-	-	CDCl_3_	[181]
**56**	61.9	62.3	61.8	61.6	-	-	-	55.3	-	-	CDCl_3_	[45]
**57**	-	61.7	61.3	61.9	61.5	-	-	55.3	-	-	DMSO-*d*_6_	[164]
**58**	-	61.8	61.9	61.6	62.2	-	56.0	-	-	-	CDCl_3_	[182]
**59**	59.2	61.7	61.3	61.8	61.5	-	-	55.3	-	-	DMSO-*d*_6_	[164]
**60**	59.9	56.4	-	56.6	61.5	56.0	-	-	55.9	-	CDCl_3_	[32]
**61**	61.4	56.5	-	56.4	59.9	-	56.0	55.9	-	-	CDCl_3_	[178]
**62**	60.0	-	56.3	61.6	62.2	56.1	-	-	56.0	-	CDCl_3_	[32]
**63**	-	61.9	55.6	61.8	55.7	-	61.5	61.4	-	-	DMSO-*d*_6_	[31]
**64**	-	61.1	61.6	62.2	-	-	56.4	56.4	56.4	-	CDCl_3_	[183]
**65**	-	61.0	-	56.5	61.4	-	55.9	56.1	55.9	-	CDCl_3_	[183]
**66**	-	62.4	62.0	61.9	61.8	-	56.1	55.8	-	-	CDCl_3_	[181]
**67**	59.7	-	60.6	61.8	61.5	-	55.4	55.7	-	-	DMSO-*d*_6_	[34]
**68**	-	-	62.0	61.1	61.7	-	56.3	61.1	56.3	-	CDCl_3_	[184]
**69**	61.6	62.3	61.8	-	61.7	-	56.0	55.9	-	-	CDCl_3_	[44]
**70**	61.9	62.3	61.8	61.6	-	-	55.9	55.8	-	-	CDCl_3_	[45]
**71**	61.5	63.7	59.3	61.6	61.4	-	56.3	55.4	-	-	DMSO-*d*_6_	[31]

**Table 12:** Chemical structure of polymethoxyflavanones (**72–77**) isolated in genus *Citrus*.

No.	Name	R_1_	R_2_	R_3_	R_4_	R_5_	R_6_	References
**72**	5,6,7,4′-Tetramethoxyflavanone	OMe	OMe	OMe	H	H	OMe	[34]
**73**	5,6,7,8,4′-Pentamethoxyflavanone	OMe	OMe	OMe	OMe	H	OMe	[185]
**74**	5,6,7,3′,4′-Pentamethoxyflavanone	OMe	OMe	OMe	H	OMe	OMe	[100]
**75**	5,7,8,3′,4′-Pentamethoxyflavanone	OMe	H	OMe	OMe	OMe	OMe	[186]
**76**	5-Hydroxy-6,7,8,3′,4′-pentamethoxyflavanone (5-Demethylcitromitine)	OH	OMe	OMe	OMe	OMe	OMe	[34]
**77**	5,6,7,8,3′,4′-Hexamethoxyflavanone (Citromitin)	OMe	OMe	OMe	OMe	OMe	OMe	[186]

**Table 13:** ^1^H-NMR and ^13^C-NMR of polymethoxyflavanones’ (**72**–**77**) skeleton isolated in *Citrus* genus.

Compound	72		73		74		75		76		77	
Solvent	DMSO-*d_6_*	CDCl_3_	CDCl_3_		CDCl_3_		DMSO-*d*_6_	CDCl_3_	CDCl_3_		CDCl_3_	
**Position**	δC	δH (J in Hz)	δC	δH (J in Hz)	δC	δH (J in Hz)	δC	δH (J in Hz)	δC	δH (J in Hz)	δC	δH (J in Hz)
**2**	79.0	5.34 (dd, 13.5, 3.0)	-	5.40 (dd, 13.2, 2.8)	79.4	5.34 (dd, 13.4, 2.7)	79.0	5.35 (dd, 13.0, 3.0)	78.7	5.40 (dd, 13.0, 3.0)	78.0	5.40 (dd, 13.0, 3.0)
**3**	45.3	3.02 (dd, 16.5, 13.5) 2.75 (dd, 16.5, 3.0)	-	3.03 (dd, 16.8, 13.2) 2.88 (dd, 16.8, 2.8)	45.5	3.03 (dd, 16.7, 13.4) 2.76 (dd, 16.7, 2.7)	45.6	3.02 (dd, 17.5, 13.0) 2.75 (dd, 17.5, 3.0)	42.1	3.09 (dd, 17.0, 13.0) 2.91 (dd, 17.0, 3.0)	45.6	3.05 (dd, 17.5, 13.0) 2.84 (dd, 17.5, 3.0)
**4**	189.3	-	-	-	189.4	-	189.2	-	198.2	-	190.2	-
**5**	156.7	-	-	-	154.2	-	156.2	-	150.8	-	151.2	-
**6**	137.5	-	-	-	137.5	-	89.5	-	133.4	-	139.5	-
**7**	160.1	-	-	-	159.4	-	157.7	-	154.9	-	154.7	-
**8**	97.5	6.31 (s)	-	-	96.4	-	132.0	-	132.8	-	141.6	-
**9**	160.0	-	-	-	159.7	-	156.8	-	148.7	-	150.5	-
**10**	109.4	-	-	-	109.1	-	107.5	-	103.9	-	112.0	-
**1′**	131.5	-	-	-	131.1	-	131.6	-	130.9	-	131.4	-
**2′**	1128.8	7.39 (d, 9.0)	-	7.01 (m)	109.4	6.99 (d, 1.8)	114.7	7.02 (d, 2.5)	110.5	7.00 (d, 2.5)	114.3	7.02 (d, 2.5)
**3′**	114.5	6.95 (d, 9.0)	-	-	149.2	-	147.0	-	149.1	-	146.7	-
**4′**	154.1	-	-	-	149.4	-	148.2	-	150.4	-	148.1	-
**5′**	114.5	6.95 (d, 9.0)	-	6.90 (d, 8.8)	111.2	6.90 (d, 8.4)	112.1	6.90 (d, 9.0)	111.6	6.90 (d, 9.0)	112.1	6.90 (d, 9.0)
**6′**	128.8	7.39 (d, 9.0)	-	7.01 (m)	118.8	7.00 (dd, 8.4, 1.8)	118.0	7.00 (dd, 9.0, 2.5)	119.1	6.99 (dd, 9.0, 2.5)	118.0	7.00 (dd, 9.0, 2.5)
**OMe-5**	61.9	3.94 (s)	-	3.90 (s)	61.3	3.91 (s)	56.5	3.92 (s)	-	-	62.0	3.90
**OMe-6**	61.5	3.83 (s)	-	4.06 (s)	56.1	3.91 (s)	-	-	60.6	4.09 (s)	61.7	4.05 (s)
**OMe-7**	56.9	3.87 (s)	-	3.90 (s)	61.6	3.82 (s)	61.4	3.82 (s)	61.2	3.80 (s)	61.8	3.82 (s)
**OMe-8**	-	-	-	3.86 (s)	-	-	56.5	3.95 (s)	61.0	3.86 (s)	56.1	3.88 (s)
**OMe-3′**	-	-	-	-	55.9	3.91 (s)	56.1	3.87 (s)	55.6	3.91 (s)	60.8	3.89 (s)
**OMe-4′**	55.9	3.83 (s)	-	3.86 (s)	55.9	3.91 (s)	56.0	3.90 (s)	55.6	3.91 (s)	56.0	3.85 (s)

**Table 14:** Chemical structures of polymethoxychalcones (**78**–**79**) occurring in *Citrus* genus.

No.	Name	R_1_	R_2_	R_3_	R_4_	R_5_	R_6_	R_7_
**78**	2′-Hydroxy-3,4,4′,5′,6′- pentamethoxychalcone	OH	H	OMe	OMe	OMe	OMe	OMe
**79**	2′-Hydroxy-3,4,3′,4′,5′,6′-hexamethoxychalcone	OH	OMe	OMe	OMe	OMe	OMe	OMe

**Table 15:** ^1^H-NMR and ^13^C-NMR data of isolated polymethoxychalcones (**78**-**79**) in *Citrus* genus.

Compounds	^1^H-NMR (CDCl_3_)	^13^C-NMR (DMSO-*d_6_*)	References
**78**	3.71 (s, 3H), 3.82 (s, 3H), 3.83 (s, 3H), 3.85 (s, 6H), 6.38 (s, 1H, H-3′), 7.02 (d, J = 8 Hz, 1H), 7.29 (dd, J = 8 Hz, 2 Hz, 1H), 7.32 (d, J = 2 Hz, 1H), 7.45 (d, J = 16 Hz, 1H), 7.57 (d, J = 16 Hz, 1H), 12.38 (s, 1H, 2′-OH)	55.5, 55.6, 56.0, 60.6, 61.5, 96.4, 110.6, 110.8, 111.7, 122.8, 125.0, 127.4, 134.6, 143.9, 149.0, 151.2, 153.0, 157.5, 157.8, 192.6	[34]
**79**	3.74 (s, 3H), 3.75 (s, 3H), 3.77 (s, 3H), 3.80 (s, 3H), 3.81 (s, 3H), 3.93 (s, 3H), 7.00 (d, J = 9 Hz, 1H,), 7.16 (d, J = 16 Hz, 1H), 7.27 (dd, J = 9 Hz, 3 Hz, 1H), 7.34 (d, J = 3 Hz, 1H), 7.40 (d, J = 16 Hz, 1H), 10.04 (s, 1H, 2′-OH)	55.6, 60.8, 60.9, 61.0, 61.5, 110.7, 111.6, 116.7, 123.2, 125.9, 127.2, 137.2, 138.6, 144.8, 145.7, 147.1, 148.9, 149.0, 151.2, 192.9	[34]

**Table 16:** Glycosylated flavones (**80**–**83**) extracted from *Citrus* genus.

No.	80		81		82		83	
Ref.	[181]		[181]		[187]		[187]	
Solvent	DMSO-*d*_6_		DMSO-*d*_6_		CD_3_OD-*d*_4_		DMSO-*d*_6_	
Position	δ_C_	δ_H_ (J in Hz)	δ_C_	δ_H_ (J in Hz)	δ_C_	δ_H_ (J in Hz)	δ_C_	δ_H_ (J in Hz)
**2**	151.0	-	151.0	-	157.6	-	156.3	-
**3**	146.2	-	146.2	-	134.4	-	133.2	-
**4**	172.2	-	172.2	-	178.5	-	177.7	-
**5**	143.5	-	143.5	-	157.1	-	156.3	-
**6**	137.4	-	137.4	-	99.3	6.29 (brs)	95.7	6.57 (s)
**7**	151.0	-	151.0	-	157.9	-	158.0	-
**8**	135.5	-	135.5	-	128.2	-	128.3	-
**9**	148.1	-	148.1	-	149.4	-	148.7	-
**10**	114.2	-	114.2	-	104.7	-	104.2	-
**1′**	122.7	-	122.7	-	122.1	-	121.2	-
**2′**	111.6	7.82 (d, 2.0)	111.5	7.83 (d, 2.0)	113.3	7.97 (brs)	115.2	7.63 (brs)
**3′**	147.3	-	147.3	-	147.5	-	144.9	-
**4′**	153.6	-	153.6	-	150.1	-	147.7	-
**5′**	112.5	7.13 (d, 9.0)	112.5	7.12 (d, 9.0)	115.2	6.94 (d, 8.0)	116.0	6.86 (d, 8.0)
**6′**	121.8	7.71 (dd, 9.0, 2.0)	121.7	7.70 (dd, 9.0, 2.0)	123.1	7.73 (brd, 8.0)	121.7	7.65 (dd, 2.0, 8.0)
**OMe**	61.9, 61.8, 61.5, 61.4, 55.7, 55.6	4.03 (s) 3.95 (s) 2× 3.86 (s) 3.84 (s) 3.82 (s)	61.9, 61.7, 61.5, 61.4, 55.7, 55.6	4.02 (s) 3.95 (s) 2× 3.85 (s) 3.84 (s) 3.82 (s)	61.2 (8-OMe) 55.8 (3′-OMe)	3.94 (s, 3H, 8-OMe) 3.98 (s, 3H, 3′-OMe)	61.1 (8-OMe) 56.5 (3′-OMe)	3.81 (s, 3H, 8-OMe) 3.90 (s, 3H, 3′-OMe)
**1″**	101.0	-	101.0	-	103.0	5.36 (d, 7.5)	100.9	5. 46 (d, 7.0)
**2″**	74.3	-	74.6	-	74.9	3.53 (m)	74.0	3.10-3.40 (m)
**3″**	74.3	-	74.3	-	77.0	3.39 (m)	76.4	3.10-3.40 (m)
**4″**	70.1	-	70.2	-	70.8	3.33 (m)	69.9	3.10-3.40 (m)
**5″**	76.4	-	76.4	-	75.0	3.50 (m)	77.5	3.08 (m)
**6″**	62.8	-	63.3	-	63.6	4.14 (dd, 1H, 3.0, 11.0) 4.25 (dd, 1H, 1.0, 11.0)	60.9	3.40 (m, 1H) 3.57 (brd, 1H, 11.5)
**1** **‴**	170.3	-	170.7	-	171.3	-	-	-
**2** **‴**	47.0	2.14 (d, 1H, 13.0 Hz) 2.09 (d, 1H, 13.0 Hz)	45.3	2.39 (2H, m)	45.2	2.52 (2H, m)	-	-
**3** **‴**	68.5	-	68.7	-	69.7	-	-	-
**4** **‴**	46.7	1.86 (d, 1H, 15.0 Hz) 2.06 (d, 1H, 15.0 Hz)	45.0	2.28 (2H, m)	45.1	2.52 (2H, m)	-	-
**5** **‴**	175.7	-	170.0	-	171.5	-	-	-
**6** **‴**	27.9	0.87 (s, 3H)	27.1	1.02 (s, 3H)	26.9	1.23 (s, 3H)	-	-
**5** **‴−OMe**	-	-	50.8	3.48 (s)	-	-	-	-

**Table 17:** Glycosylated flavones (**84**–**87**) extracted from *Citrus* genus.

No.	84	85	86		87			
Ref.	[188]	[188]	[188]		[189]			
Solvent	CD_3_OD-*d*_4_	CD_3_OD-*d*_4_	DMSO-*d*_6_		Pyridine-*d*_5_			
Position	δ_H_ (J in Hz)	δ_H_ (J in Hz)	δ_C_	δ_H_ (J in Hz)	δ_C_	δ_H_ (J in Hz)	δ_C_	δ_H_ (J in Hz)
**2**	-	-	156.8	-	163.8	-	165.0	-
**3**	-	-	135.5	-	104.7	6.99 (s)	103.9	7.02 (s)
**4**	-	-	170.1	-	183.8	-	183.5	-
**5**	-	-	147.1	-	150.0	-	149.2	-
**6**	-	-	133.0	-	133.2	-	137.4	-
**7**	-	-	150.1	-	152.8	-	150.0	-
**8**	-	-	131.3	-	129.4	-	134.1	-
**9**	-	-	144.3	-	146.6	-	146.3	-
**10**	-	-	106.5	-	104.4	-	108.1	-
**1′**	-	-	120.6	-	126.1	-	122.6	-
**2′**	7.87 (d, 2.0)	7.98 (d, 2.0)	113.0	7.84 (d, 2.0)	111.1	7.61 (d, 2.2)	110.6	7.67 (d, 2.2)
**3′**	-	-	152.4	-	150.6	-	149.6	-
**4′**	-	-	148.2	-	151.1	-	152.8	-
**5′**	6.96 (d, 9.0)	6.93 (d, 8.0)	115.5	6.97 (d, 8.0)	117.0	7.68 (d, 8.6)	117.3	7.30 (d, 8.2)
**6′**	7.85 (dd, 9.0, 2.0)	7.70 (dd, 8.0, 2.0)	122.5	7.64 (dd, 8.0, 2.0)	120.7	7.87 (dd, 8.6, 2.2)	121.6	7.77 (dd, 8.2, 2.2)
**−OMe**	4.08 (s) 3.97 (s) 2x 3.92 (s) 3.90 (s) 3.89 (s)	4.04 (s) 3.91 (s) 3.87 (s) 3.83 (s)	61.7 61.4 60.5 55.6	4.02 (s) 3.91 (s) 3.87 (s) 3.83 (s)	61.6 60.6 56.3	4.09 (s) 4.00 (s)	62.4 61.2 56.2	4.24 (s) 4.21 (s)
**1″**	4.88 (d, 7.0)	5.46 (d, 7.0)	100.9	5.45 (d, 8.0)	102.3	5.73 (d, 7.3)	104.5	6.15 (d, 7.3)
**2″**	-	-	74.2	2.18-2.33 (m, 2H)	74.7	-	75.7	-
**3″**	-	-	76.2		78.3	-	78.2	-
**4″**	-	-	70.0	2.18-2.33 (m, 2H)	71.5	-	71.4	-
**5″**	-	-	74.2		75.7	-	75.9	-
**6″**	-	-	63.0	1.01 (s, 2H)	64.7	5.02 (dd, 12.0, 2.2) 4.74 (dd, 12.0, 6.5)	64.4	4.99 (dd, 12.0, 1.0) 4.81 (dd, 12.0, 5.2)
**1** **‴**	-	-	173.7	-				
**2** **‴**	-	-	45.3	-				
**3‴**	-	-	68.6	-				
**4** **‴**	-	-	45.6	-				
**5** **‴**	-	-	177.6	-				
**6** **‴**	-	-	27.0	-				
**COO-**	-	-	-	-	171.6	-	171.6	-
	-	-	-	-	δ_C_		δ_H_ (J in Hz)	
**−CH_2−_**	-	-	-	-	46.3		2.88 (d, 14.6) 2.99 (d, 14.6)	
**−CH_2−_**	-	-	-	-	46.5		2.97 (d, 14.5) 3.04 (d, 14.5)	
**−COH**	-	-	-	-	70.1		-	
**Me**	-	-	-	-	28.0		1.57 (s)	

**Table 18:** Glycosylated flavones (**88**–**91**) extracted from *Citrus* genus.

No.	88		89		90		91	
Ref.	[189]		[189]		[189]		[189]	
Solvent	Pyridine-*d*_5_		Pyridine-*d*_5_		Pyridine-*d*_5_		Pyridine-*d*_5_	
Position	δ_C_	δ_H_ (J in Hz)	δ_C_	δ_H_ (J in Hz)	δ_C_	δ_H_ (J in Hz)	δ_C_	δ_H_ (J in Hz)
**2**	163.8	-	163.8	-	165.3	-	164.8	-
**3**	104.6	6.98 (s)	104.8	6.99 (s)	104.1	7.01 (s)	103.9	6.99 (s)
**4**	183.3	-	183.3	-	183.7	-	183.4	-
**5**	150.0	-	150.0	-	150.1	-	149.2	-
**6**	133.2	-	133.2	-	137.6	-	137.3	6.57 (s)
**7**	152.8	-	152.8	-	150.1	-	150.0	-
**8**	129.4	-	129.4	-	134.2	-	133.9	-
**9**	146.6	-	146.6	-	146.4	-	146.2	-
**10**	104.5	-	104.4	-	108.2	-	107.8	-
**1′**	125.8	-	126.1	-	122.8	-	122.6	-
**2′**	111.2	7.64 (d, 1.4)	111.2	7.61 (d, 2.0)	110.9	7.69 (d, 2.0)	110.6	7.67 (d, 2.2)
**3′**	150.5	-	150.7	-	150.2	-	150.1	-
**4′**	151.3	-	151.2	-	152.9	-	152.9	-
**5′**	116.7	7.68 (d, 8.5)	117.1	7.74 (d, 8.4)	117.4	7.31 (d, 8.4)	117.3	7.32 (d, 8.6)
**6′**	120.6	7.71 (dd, 8.5, 1.4)	120.7	7.91 (dd, 8.4, 2.0)	121.7	7.77 (dd, 8.4, 2.0)	121.5	7.78 (dd, 2.2, 8.5)
**OMe**	61.5 60.5 56.4	4.03 (s) 4.00 (s)	61.6 60.6 56.4	4.10 (s) 4.00 (s)	62.4 61.3 56.4	4.28 (s) 4.23 (s)	62.2 61.1 56.2	4.21 (s) 4.19 (s)
**1″**	102.2	5.83 (d, 7.1)	102.8	5.74 (d, 6.8)	104.6	6.15 (d, 7.2)	104.5	6.28 (d, 7.0)
**2″**	74.9	-	74.8	-	75.7	-	75.9	-
**3″**	78.6	-	78.5	-	78.4	-	78.2	-
**4″**	71.5	-	71.6	-	71.5	-	71.7	-
**5″**	79.1	-	75.8	-	76.0	-	79.2	-
**6″**	62.6	4.59 (dd, 12.1, 2.1)	64.8	5.14 (dd, 12.1, 1.6) 4.77 (dd, 12.1, 6.8)	64.5	4.99 (d, 11.6) 4.80 (dd, 11.6, 5.6)	62.7	4.54 (dd, 11.8, 2.6)
**COO-**	-	-	171.7	-	171.8	-	-	-
**COOH**	-	-	174.7	-	174.8	-	-	-
**−CH_2−_**	-	-	46.4	3.13 (d, 14.1) 3.32 (d, 14.1)	46.5	2,99 (d, 14.5) 3.08 (d, 14.5)	-	-
**−CH_2−_**	-	-	46.7	3.14 (d, 15.3) 3.20 (d, 15.3)	46.5	3.01 (d, 15.0) 3.07 (d, 15.0)	-	-
**−COH**	-	-	70.1	-	70.1	-	-	-
**Me**	-	-	28.3	1.74 (s)	28.1	1.63 (s)	-	-
